# Precursor-Stage Electronic and Stacking-Coherence Modulation of Layered PbI_2_ via In Situ Ti_3_C_2_T_*x*_ MXene Incorporation

**DOI:** 10.1021/acsomega.6c02335

**Published:** 2026-06-19

**Authors:** Dagoberto Cabrera-German, Luis Armando Urias-Zavala, Lorenzo Fuentes-Ríos, Guillermo Suárez-Campos, Jeisson Solis-Mosquera, Martín A. Ruiz-Molina, Rubén Orlando Grijalva-Saavedra, Manuel Quevedo-Lopez, Mérida Sotelo-Lerma

**Affiliations:** † Departamento de Investigación en Polímeros y Materiales, 27813 Universidad de Sonora, Blvd Luis Encinas y Rosales s/n Sonora, Hermosillo 83000, México; ‡ Departamento de Investigación en Física, Universidad de Sonora, Blvd. Luis Encinas y Rosales s/n, C.P., 83000 Hermosillo, Sonora, Mexico; § Materials Science and Engineering Department, 12335 University of Texas at Dallas, 800 West Campbell Road, Richardson, Texas 75080, United States

## Abstract

MXene composite thin films, PbI_2_–Ti_3_C_2_T_*x*_, were fabricated by sequential dynamic spin-coating using a water/ethanol precursor system that enables in situ MXene incorporation while avoiding strongly coordinating solvents. This study addresses a central gap in halide precursor engineering: PbI_2_ is commonly treated as a transient phase before perovskite conversion, although its local structure and electronic environment can influence subsequent material formation. Here, we show that Ti_3_C_2_T_*x*_ incorporation modifies PbI_2_ at the precursor level without disrupting the 2H–PbI_2_ framework. Profilometry shows that the average film thickness remains nearly constant across the composition series, whereas roughness and surface morphology evolve with MXene loading. Scanning electron microscopy with energy-dispersive X-ray spectroscopy (SEM-EDS) identifies Ti-rich clustered regions associated with Pb–I-containing material, supporting a growth-mediated incorporation pathway. X-ray diffraction confirms preservation of the 2H structure, while the PbI_2_ (001) basal-envelope line shape evolves with composition, consistent with changes in stacking-related environments. Raman spectroscopy shows preservation of the PbI_2_ vibrational fingerprint together with mode-selective perturbations. X-ray photoelectron spectroscopy (XPS) reveals statistically significant changes in the Pb 4f-I 3d core-level separation, indicating modification of the local Pb–I electrostatic and chemical environment rather than uniform charging or oxidation-state transformation. Optical measurements show a preserved absorption edge, while photoluminescence quenching and photoelectrical response indicate MXene-associated interfacial deactivation and carrier redistribution. Together, these results show that PbI_2_ can be treated as an engineerable precursor whose local morphology, stacking-related order, and electronic environment can be tuned before conversion into perovskite.

## Graphical abstract

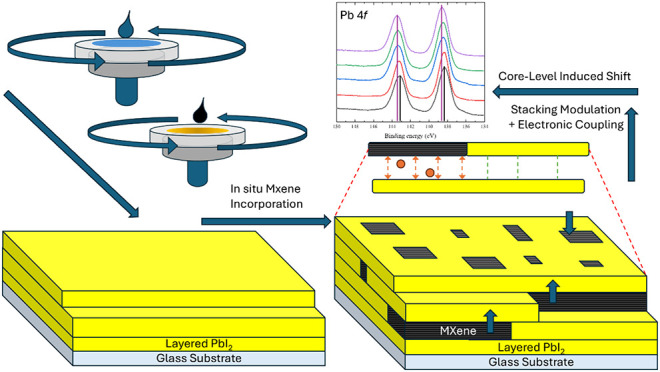

## 1 Introduction

Lead iodide (PbI_2_) occupies a central position in halide thin-film processing because it is both a layered wide-bandgap semiconductor and the dominant inorganic precursor in two-step metal-halide perovskite fabrication.^1–4^ In sequential deposition routes,^5,6^ the first-deposited PbI_2_ layer does not act only as a passive lead reservoir. Its morphology, crystallinity,^7–9^ stoichiometry, and local chemical environment influence ion infiltration, conversion completeness, defect formation, and the final organization of the halide film. Therefore, controlling PbI_2_ before conversion has become an important strategy for improving downstream perovskite film formation and device behavior.^5,6^

The chemistry of PbI_2_ formation depends strongly on the precursor solvent environment.^10–13^ Strongly coordinating aprotic solvents such as *N*,*N*-dimethylformamide (DMF)^14^ and dimethyl sulfoxide (DMSO)^15^ stabilize Pb-containing adducts and reshape nucleation pathways by altering the balance between Pb-solvent and Pb–I interactions.^15,16^ Although these coordination effects can benefit morphology control, they also complicate the interpretation of how additives affect PbI_2_ itself. Moreover, DMF-based processing introduces handling and toxicity concerns,^17–19^ motivating interest in alternative solvent systems. In this context, water/ethanol-based processing provides a useful hydroalcoholic route for evaluating Pb–I film formation outside the conventional DMF/DMSO-dominated solvent framework, while retaining compatibility with solution processing.

In parallel, two-dimensional MXenes, especially Ti_3_C_2_T_*x*_, provide another route to modify halide materials because they combine high electrical conductivity, tunable work function, solution processability, and surface terminations such as −O, −OH, and −F.^20–22^ These features have motivated their use in perovskite solar cells as absorber additives,^4,23^ charge-transport modifiers,^24–26^ interfacial layers, electrodes, and precursor additives. However, most MXene-halide studies remain centered on the final perovskite absorber or device architecture.^20,23,27–31^ Even the closest studies,^4,23^ where Ti_3_C_2_T_*x*_ is introduced into a PbI_2_-containing step, primarily aim to improve perovskite conversion, grain growth, carrier extraction, or device stability rather than to isolate the PbI_2_:Ti_3_C_2_T_*x*_ film as an independent material system.^4,23^

Recent MXene-composite studies beyond perovskite photovoltaics further show that local chemical modification, interlayer/interfacial engineering, and hybridization with conductive or functional phases can strongly affect morphology, charge redistribution, structural stability, and electronic or photoactive response. For example, Ti_3_CNT_*x*_-based and V_2_CT_*x*_-based hybrids have used conductive components, metal-ion intercalation, or electrostatic self-assembly to tune charge-transfer behavior, interlayer spacing, and functional performance.^32–34^ MXene-derived and free-standing MXene films have also been explored for electronic and memory-device applications.^35–37^ These studies highlight a broader principle relevant to the present work: MXene-derived regions can act as chemically active interfacial components rather than passive fillers. However, these reports primarily address catalysis, energy storage, or electronic memory systems, and do not resolve how Ti_3_C_2_T_*x*_ modifies PbI_2_ itself during halide precursor-film formation.

This leaves a specific gap. Although PbI_2_-stage additive engineering has been shown to influence later perovskite crystallization,^38^ the direct effect of Ti_3_C_2_T_*x*_ incorporation during PbI_2_ film formation remains insufficiently resolved, particularly under sequential hydroalcoholic deposition conditions. The missing question is not whether MXenes can improve perovskite devices or other functional platforms, but how MXene-derived regions modify PbI_2_ itself before conversion into perovskite. Specifically, it remains unclear how Ti_3_C_2_T_*x*_ incorporation affects local morphology, Pb–I chemical-state distribution, stacking-related diffraction signatures, vibrational response, near-surface electronic environment, optical behavior, and photoelectrical response while preserving the native layered PbI_2_ framework.

Here, we address this gap by preparing PbI_2_:Ti_3_C_2_T_*x*_ composite thin films through sequential dynamic spin-coating using a water/ethanol precursor system. Ti_3_C_2_T_*x*_ is introduced during PbI_2_ film formation through the iodide precursor solution, enabling direct comparison across nominal MXene-to-Pb ratios. By combining profilometry, scanning electron microscopy with energy-dispersive X-ray spectroscopy (SEM-EDS), X-ray diffraction (XRD), Raman spectroscopy, X-ray photoelectron spectroscopy (XPS), optical absorption, photoluminescence (PL), and current density–voltage (*J*–*V*)/photoresponse, we evaluate how MXene incorporation modifies the PbI_2_ film while preserving the 2H–PbI_2_ framework. The resulting analysis supports a growth-mediated incorporation interpretation in which MXene-derived Ti-rich regions associate with Pb–I-containing material and modify local crystallite organization, stacking-related environments, and near-surface Pb–I electronic structure without inducing bulk phase transformation or systematic lattice expansion.

## 2 Experimental Section

### 2.1 Materials

Lead­(II) nitrate (Pb­(NO_3_)_2_, 99.99%), potassium iodide (KI, 99%), ethanol (96%), isopropyl alcohol (99.5%), and ammonium citrate dibasic were purchased from Sigma-Aldrich. Ti_3_C_2_T_*x*_ MXene multilayer nanoflakes were acquired from MSE supplies. All chemicals were used as received without further purification. Deionized water (18.2 MΩ·cm) was used throughout all experiments.

### 2.2 Precursor Solution Preparation

#### 2.2.1 Lead Precursor (R-Pb)

A 0.25 M Pb^2+^ stock solution was prepared by dissolving Pb­(NO_3_)_2_ in 5.00 mL of deionized water, followed by the addition of 5.00 mL of ethanol (96%). For spin-coating, this stock solution was diluted 1:10 with ethanol to obtain the working lead precursor (R-Pb, 0.025 M).

#### 2.2.2 Iodide Precursor (R-I)

An iodide stock solution (0.30 M KI) was prepared in a total volume of 14.00 mL consisting of 5.00 mL deionized water, 5.00 mL ethanol, and 4.00 mL of 0.025 M ammonium citrate. For spin-coating, the working iodide precursor (R-I, 0.03 M) was obtained by diluting the stock solution 1:10 with ethanol.

Preliminary optimization experiments demonstrated that this iodide concentration enabled the deposition of continuous and uniform films. Higher iodide concentrations produced nonuniform coatings and poor film formation. Therefore, all subsequent depositions were performed using the optimized R-I concentration.

#### 2.2.3 Preparation of Iodide–MXene Dispersions

Ti_3_C_2_T_*x*_ MXene was dispersed in ethanol by ultrasonication to obtain a stock dispersion with a concentration of 10 mg/mL. Controlled volumes of the MXene dispersion were added to separate R-I solutions to achieve nominal MXene-to-Pb mass ratios of 0, 0.25, 0.45, 0.65, and 1.00 relative to the Pb content introduced from the R-Pb precursor. These additions yielded final MXene concentrations of 0, 1.2, 1.9, 2.5, and 3.4 mg/mL in the modified R-I solutions. All mixtures were ultrasonicated for 15 min and maintained under continuous magnetic stirring during the deposition process.

### 2.3 Film Deposition by Spin-Coating

Glass substrates (LAUKA brand slides; 2.5 × 2.5 cm^2^) were sequentially cleaned with Alconox detergent, deionized water, and ethanol, followed by a 15 min UV–ozone treatment to improve surface wettability. Thin films were deposited using a two-step dynamic spin-coating process performed at 3000 rpm.

#### 2.3.1 Deposition of R-Pb Solution

The substrate was accelerated to 3000 rpm and allowed to stabilize. Then, 200 μL of the R-Pb solution was dispensed onto the rotating substrate. The film was spun at 3000 rpm for 20 s to promote uniform spreading, after which rotation was stopped.

#### 2.3.2 Deposition of R-I Solution (With or Without MXene)

The substrate was accelerated again to 3000 rpm. Subsequently, 200 μL of the R-I solution, either pristine or containing MXene, was dispensed onto the rotating substrate. The film was spun for 20 s before stopping the rotation. One complete deposition cycle consisted of sequential deposition of 200 μL of R-Pb followed by 200 μL of R-I. Figure 1 shows a schematic representation of the experimental procedure. This sequence produced one PbI_2_ layer. The two-step procedure was repeated nine additional times to obtain films composed of 10 stacked layers. The films were labeled as s00, s25, s45, s65, and s100, corresponding to nominal MXene-to-Pb mass ratios of 0, 0.25, 0.45, 0.65, and 1.00, respectively.

**Figure 1:**
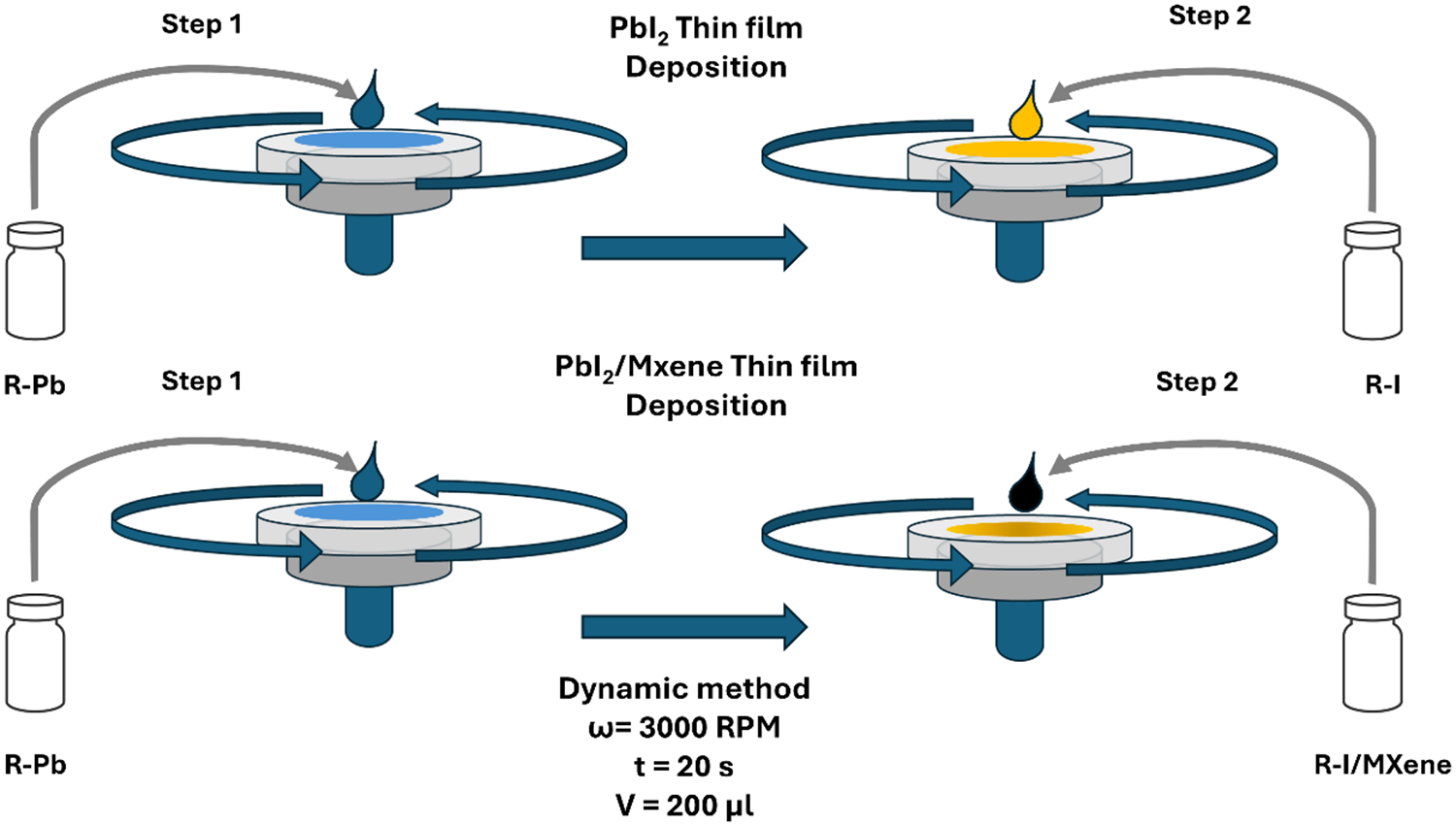
Schematic of the sequential dynamic spin-coating process used to fabricate PbI_2_ and PbI_2_–MXene thin films. A two-step deposition cycle (3000 rpm, 20 s, 200 μL per solution) consists of R-Pb deposition followed by R-I (pristine) or R-I/MXene. Repetition of the cycle yields multilayer films with in situ MXene incorporation.

In addition, a pristine Ti_3_C_2_T_*x*_ reference film was prepared by spin-coating the 10 mg/mL MXene dispersion in 96% ethanol onto a cleaned glass substrate, without addition of the R-Pb or R-I precursor solutions. This reference film was used only for comparative SEM, XPS, and Raman measurements.

### 2.4 Characterization

Surface morphology was examined using a JEOL JSM-5410LV system. Elemental composition was analyzed by energy-dispersive X-ray spectroscopy (EDS) using a Bruker Quantax 200 detector operated at 3 kV. Film thickness was determined using an Ambios XP-200 profilometer by performing one-dimensional line scans from 0 to 8 mm, with an applied force of 3 mg and a step duration of 1 min. More details can be found in the Supporting Information (SI) file.

XRD patterns were acquired using a Rigaku SmartLab diffractometer with Cu Kα radiation (λ = 1.5406 Å) in a thin-film configuration with a fixed incident angle of ω = 0.2°. Patterns were collected over a 2θ range of 10–50°. The patterns were analyzed by whole-pattern profile matching with a constant scale factor using the FullProf suite.^39,40^ Peak shapes were described using the Thompson-Cox-Hastings pseudo-Voigt function, including axial-divergence correction. The reflection list was generated directly from the space group using crystallographic information from the corresponding Crystallographic Information File downloaded from the Internet crystallographic database,^41–43^ with both the Cu Kα_1_ and Kα_2_ wavelengths considered. The free variables in the profile-matching procedure were the Gaussian and Lorentzian widths, the 2θ position, and the intensity.

Raman spectra were acquired with a Thermo Scientific DXR Raman microscope using a 532 nm excitation laser operating at 5 mW. The measurements were collected using a 10× objective with a numerical aperture (NA) of 0.25 and a 25 μm confocal pinhole. The exposure and acquisition time were set to 2 s over 32 cycles.

XPS was performed using a PerkinElmer PHI 5600 ESCA System equipped with a monochromatic Al Kα X-ray source (hν = 1486.7 eV). Spectra were recorded at a pass energy of 29.35 eV with a step size of 0.125 eV in large area XPS mode with a spot size of ∼650 μm. Peak-fitting analysis for both Raman and photoelectron spectra in this work was done using a freely distributed software within the XPS OASIS platform employing state-of-the-art methods.^44–47^ The uncertainties of the free variables, including peak width, position, and intensity of the Voigt profiles, were assessed using the covariance method approach^46^ with 95% confidence, corresponding to twice the standard deviation with the appropriate uncertainty propagation. Chemical composition was calculated considering the photoelectron attenuation according to appropriate physical parameters.^48–50^

UV–Vis transmittance and reflectance spectra were measured with a Shimadzu UV-3600 spectrophotometer in the wavelength range of 300–1000 nm. PL spectra were acquired using a Spectral Products DK480 1/2 m monochromator. The samples were excited with a 405 nm laser at an irradiance of 100 mW cm^–2^, using a 0.8 mm aperture, and the emission spectra were collected under the same conditions for all samples.

Electrical measurements were performed on patterned fluorine-doped tin oxide (FTO) substrates. Current–voltage (*I*–*V*) characteristics were recorded using a four-point probe configuration with a Keithley 2450 sourcemeter.

## 3 Results and Discussion

### 3.1 Film Thickness and Roughness

The sequential dynamic spin-coating process produced macroscopically continuous PbI_2_ and PbI_2_-MXene composite films on glass substrates. As shown by the inset photographs in Figure 2 for representative samples s00 and s100, both the undoped film and the highest MXene-loading film covered the substrate surface over the inspected macroscopic area. The films remained attached to the substrates during handling, after rinsing with water from a wash bottle, and after a qualitative wet cotton-swab test, with no visible delamination. We use these observations only as practical evidence of film adherence during processing and characterization rather than as quantitative or standardized adhesion measurements.

**Figure 2:**
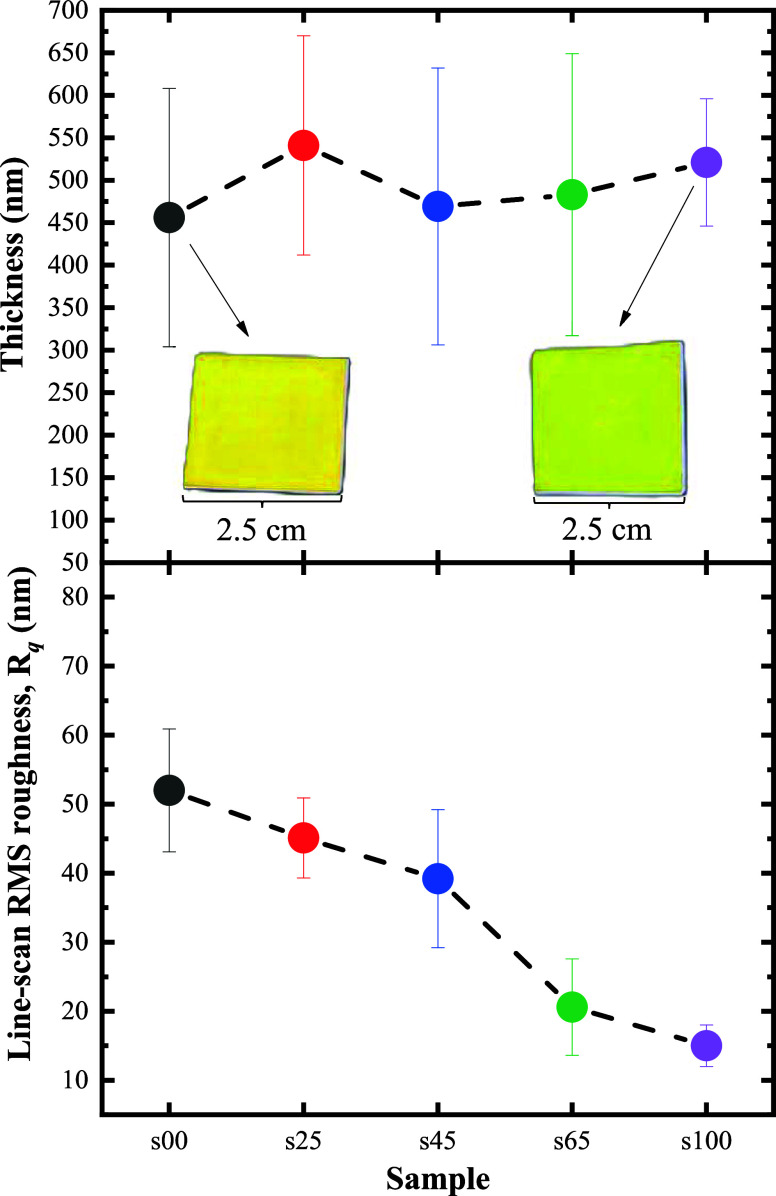
Film thickness and roughness of PbI_2_–MXene composite films as a function of nominal MXene-to-Pb mass ratio (s00–s100), measured by profilometry.

The film thickness results shown in Figure 2 demonstrate that the films maintain a nearly constant average thickness across the full composition series. All samples exhibit mean thickness values close to 500 nm, and the overlapping uncertainty intervals support that MXene does not measurably alter the overall film thickness within the experimental resolution. These results show that the sequential dynamic spin-coating process preserves a consistent film build-up despite changes in MXene loading.

From the height profiles, we also calculated the line-scan root-mean-square roughness, *R*_*q*_, from the residuals of the left and right plateau regions after independently leveling each region using its fitted linear baseline. In contrast to the thickness results, the line-scan roughness shows a clearer compositional response. Samples s00, s25, and s45 exhibit similar *R*_*q*_ values, whereas s65 and s100 show a pronounced reduction in roughness. This behavior indicates that MXene loading affects surface topography more strongly than total film build-up. In this sense, Ti_3_C_2_T_*x*_ incorporation primarily modifies the local surface leveling characteristics of the films rather than producing a resolved change in the average deposited thickness.

### 3.2 Surface Morphology

SEM micrographs (Figure 3) show a clear composition-dependent evolution of the local surface morphology after incorporation of Ti_3_C_2_T_*x*_ into the PbI_2_ matrix. The pristine film (s00) exhibits a compact surface composed of densely packed quasi-spherical grains with submicron lateral dimensions. Within the inspected field, these grains form a continuous and relatively uniform granular layer.

**Figure 3:**
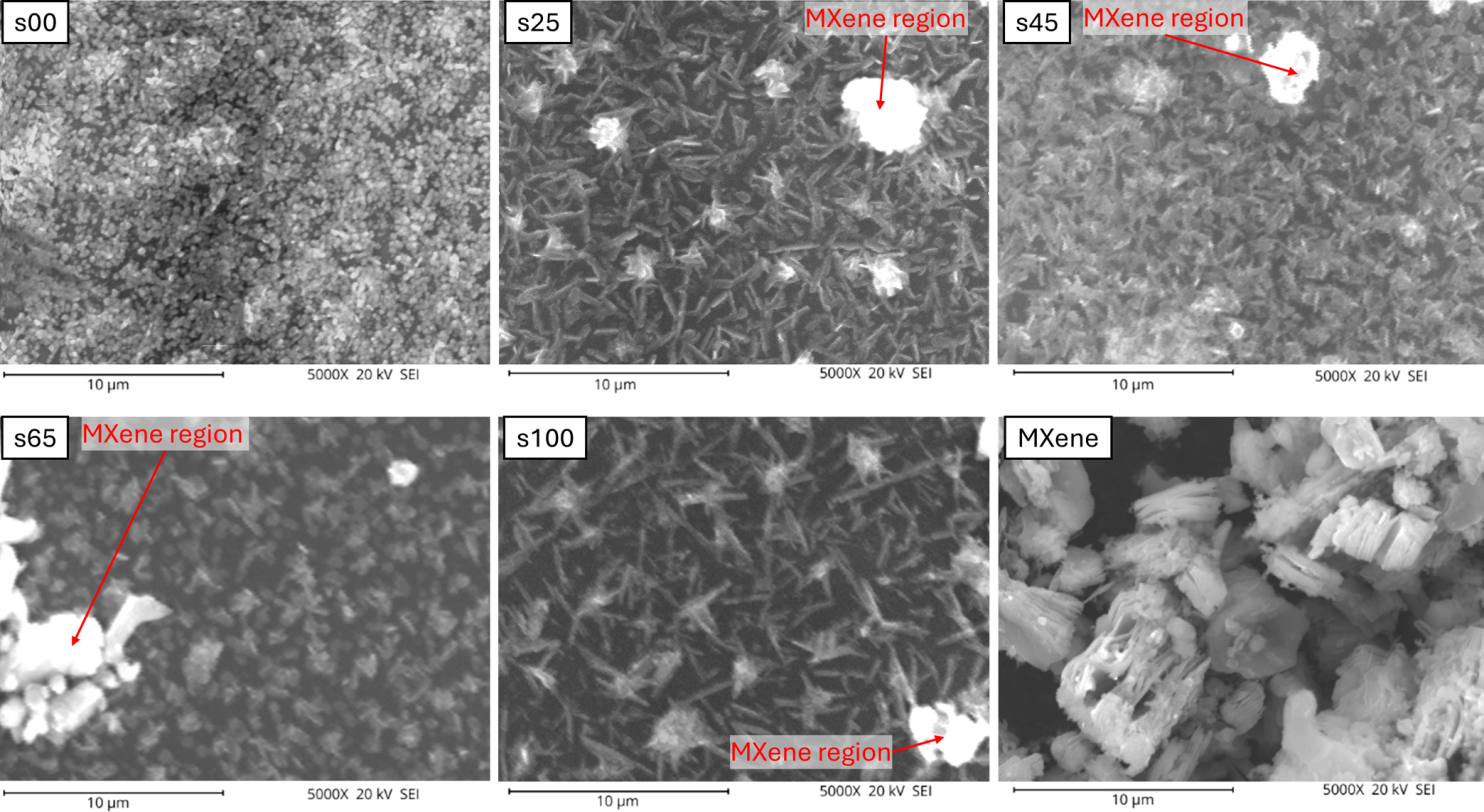
SEM micrographs (5000×, 10 μm scale bar) of PbI_2_–MXene films with increasing nominal MXene-to-Pb mass ratio: s00, s25, s45, s65, and s100. A reference micrograph of pristine Ti_3_C_2_T_*x*_ MXene is shown for comparison. MXene region labels indicate surface area regions corresponding to MXene-derived Ti-rich regions.

Upon MXene incorporation, the morphology changes significantly. In s25, the granular surface gives way to elongated platelet-like crystallites with lateral dimensions on the order of ∼1–2 μm. Several of these platelet assemblies appear to develop from localized clustered regions, indicating a substantial change in crystal habit relative to s00. At this stage, the main observable effect of MXene incorporation is the transition from a compact granular texture to a platelet-rich morphology. These results are consistent with previous reports showing that MXene-type additives can influence crystallization pathways and local crystal organization in related systems.^4,51^

The s45 sample retains the platelet-like morphology, but the surface appears more uniformly covered by these anisotropic crystallites than in s25. The platelet distribution is more even across the inspected region, giving s45 a more homogeneous platelet-rich texture while remaining clearly distinct from the compact granular morphology of s00.

The morphology changes again in s65. In this sample, the dominant surface features become shorter, broader, and less sharply defined than the platelets observed in s25 and s45, and local agglomerated regions are also present. Thus, s65 no longer resembles the well-developed elongated platelet morphology observed at intermediate loading but instead shows a different crystallite population with increased local morphological heterogeneity.

At the highest loading, s100 exhibits larger platelet aggregates and more pronounced clustered growth features in the order of ∼3–5 μm. The elongated crystallites frequently appear organized around localized centers, producing star-like or rosette-like assemblies that are larger than those observed at lower MXene contents. The pristine MXene micrograph, acquired from a spin-coated Ti_3_C_2_T_*x*_ film, is included as a morphological reference because it shows stacked lamellar agglomerates that differ from the compact granular PbI_2_ film and resemble the clustered features observed in the composite films.

To evaluate whether the bright clustered regions correspond to MXene-derived Ti-rich domains, EDS spectra were acquired from large-area regions of approximately 3000 μm^2^ and from selected small-area regions located on the bright clustered features. Representative SEM-EDS acquisition regions and corresponding spectra are provided in the Supporting Information. The elemental analysis (Table 1) was restricted to Pb, I, and Ti signals and renormalized to these elements to compare the composite-relevant composition while minimizing contributions from the glass substrate and adventitious surface species. The large-area spectra show lower Ti contents because they average both PbI_2_-rich regions and localized Ti-containing domains. In contrast, the small-area spectra collected from the bright clustered features show markedly higher Ti fractions, confirming that these regions are Ti-rich and therefore associated with MXene-derived regions.

**Table 1:** Atomic Percentages Calculated After Normalizing only the Pb, I, and Ti EDS Signals^a^

	large-area composition (∼3,000 μm^2^)			small-area composition (clustered regions ∼2–120 μm^2^)		
sample	Pb at.%	I at.%	Ti at.%	Pb at.%	I at.%	Ti at.%
s00	40.8	59.2	0.0	40.8	59.2	0.0
s25	37.4	57.3	5.3	13.3	26.1	60.6
s45	39.3	57.4	3.3	26.5	49.0	24.5
s65	24.7	35.8	39.5	5.1	9.8	85.1
s100	22.5	37.9	39.6	6.7	13.6	79.7

a Other detected elements were excluded because they may include substrate or adventitious contributions and were not used to evaluate the PbI_2_-MXene compositional distribution

The small-area EDS analysis also shows that Pb and I remain present within these Ti-rich regions. Moreover, the local I/Pb ratios in several bright regions approach the PbI_2_-like stoichiometric value more closely than the corresponding large-area averages. This result suggests that the Ti-rich clustered regions are not simply isolated MXene agglomerates, but are associated with Pb–I material. Therefore, the combined SEM-EDS evidence supports the interpretation that MXene-derived regions act as preferential sites for local PbI_2_ crystallite organization. However, because EDS provides local semiquantitative compositional information rather than time-resolved nucleation evidence, we interpret these regions as MXene-associated growth sites rather than as direct proof of the complete nucleation pathway.

Taken together, the micrographs and EDS analysis reveal a nonmonotonic morphological evolution from compact granular PbI_2_ in s00 to elongated platelet-rich surfaces in s25 and s45, followed by a second morphological transition at higher MXene loading in s65 and s100, where the crystallites become more clustered and locally heterogeneous. This evolution indicates that Ti_3_C_2_T_*x*_ modifies the crystallization behavior and surface organization of the PbI_2_ films rather than acting as an inert additive, in agreement with previous reports.^4,51^

### 3.3 Structural Analysis

XRD patterns (Figure 4, left panel) indicate that all films crystallize predominantly as hexagonal PbI_2_ (2H polytype; Powder diffraction file (PDF) #07–0235).^7^ The diffractograms show a strong reflection at 2θ ∼ 12.6°, assigned to the (001) plane, along with weaker reflections near 25.9° and 34.3° corresponding to the (101) and (102) planes, respectively. The persistence of these reflections across all compositions demonstrates that Ti_3_C_2_T_*x*_ incorporation does not disrupt the underlying 2H–PbI_2_ framework within the detection limits of XRD. No additional Pb-containing crystalline secondary phases are detected.

**Figure 4:**
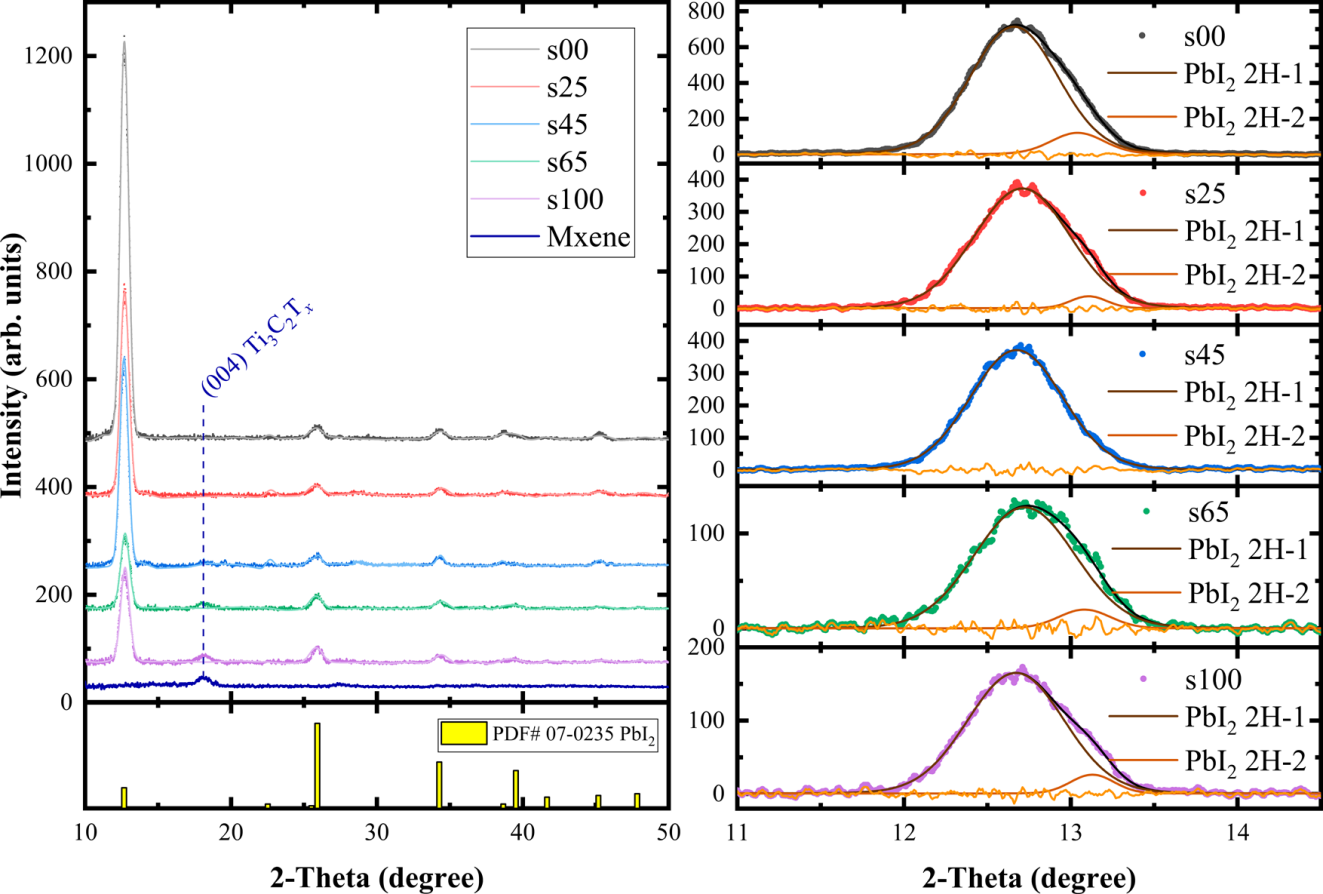
XRD analysis of PbI_2_–MXene films with increasing MXene content. The left panel shows the full diffraction patterns, with solid lines representing the whole-pattern profile-matching fits obtained from FullProf, together with the Ti_3_C_2_T_*x*_ MXene reference and the hexagonal PbI_2_ reference pattern (PDF #07–0235). The right panel shows the expanded PbI_2_ (001) region with representative two-component fits used to describe the asymmetric basal envelope.

At higher MXene incorporation (s65 and s100), a weak feature appears near 2θ ∼ 18.2°, consistent with plane (004) of the reference pattern of Ti_3_C_2_T_*x*_.^52–55^ Although of low intensity, the emergence of this reflection only at elevated nominal MXene content provides diffraction-level evidence for the presence of MXene-related regions within the composite films. The limited number and weak intensity of MXene-related reflections are consistent with low overall MXene fraction, partial exfoliation, and/or limited long-range order of the dispersed MXene domains.

The XRD data show that the relative intensity of the PbI_2_ (001) reflection decreases with MXene incorporation. This decrease likely reflects changes in the preferred [001] orientation commonly observed in chemically deposited PbI_2_ thin films grown on glass,^7^ with an additional contribution from the progressive microstructural changes induced by MXene. Because diffraction intensity depends on texture, crystallite orientation, illuminated volume, and microstructural organization, we interpret these intensity variations qualitatively rather than as direct measures of phase fraction. The SEM images show a nonmonotonic morphological evolution from compact quasi-spherical PbI_2_ grains in s00 to platelet-rich surfaces in s25 and s45, followed by more clustered and locally heterogeneous crystallite populations in s65 and s100. This morphological evolution does not necessarily imply stronger (001) diffraction intensity. Platelet-like growth can coexist with broader orientation distributions, reduced vertical registry, or preferential exposure of crystallographic planes that do not reinforce the basal (001) reflection. Therefore, the simultaneous reduction in (001) intensity and emergence of platelet-like morphology suggest that MXene incorporation modifies the orientation distribution and basal stacking coherence of PbI_2_. Similar intensity redistribution associated with texture and microstructural evolution has been reported in MXene-modified PbI_2_ systems.^4,51^

The left panel of Figure 4 shows the profile-matching results obtained using two 2H-related PbI_2_ model phases based on the hexagonal *P*-3*m*1 framework. The model phases were generated using a common in-plane lattice parameter of *a* = 4.521 Å. We use the term “model phases” to describe the fitting implementation; physically, these contributions represent two 2H-like basal-spacing components with different average *c*-axis spacings rather than as independent crystallographic phases. The calculated profiles reproduce the main features of the experimental patterns, confirming that the chemical-solution route preserves the layered 2H–PbI_2_ framework across the series.

A satisfactory whole-pattern profile-matching description can be approached using a single 2H–PbI_2_ structural model. Yet, the basal (001) reflection exhibits a pronounced asymmetric envelope that conventional axial-divergence and skew-correction terms could not satisfactorily reproduce within a single-component treatment. In addition, Rietveld refinements using standard preferred-orientation corrections, including March-Dollase-type corrections, did not reproduce the strongly textured thin-film profiles while maintaining physically coherent parameters. Stacking-fault-specific approaches, such as FAULTS- or DIFFaX-type simulations, could in principle provide a more rigorous description of layered stacking disorder because these methods explicitly model layer sequences, stacking vectors, and transition probabilities.

However, the present laboratory XRD patterns were acquired in a thin-film geometry with a fixed incident angle, which enhances surface sensitivity and can further emphasize reflections from highly oriented crystallites. Consequently, in these strongly textured PbI_2_ films, the diffraction response is dominated by the intense basal reflection, whereas the nonbasal and higher-order reflections needed to constrain stacking-disorder models remain weak. This intensity imbalance limits the independent diffraction information available to obtain a robust stacking-fault refinement or to discriminate uniquely among different faulting scenarios. Therefore, rather than reporting an underconstrained stacking-fault model, we treated the XRD response phenomenologically using a constrained two-component basal-envelope model. We interpret the fitted components as apparent basal-spacing and coherence-related contributions, not as independently refined crystallographic phases, and use them to track reproducible line-shape evolution across the MXene series.

A closer examination of the PbI_2_ (001) region, shown in the right panel of Figure 4, reveals pronounced asymmetry in all samples. The basal envelope was consistently described using two doublet contributions, where each doublet accounts for the Cu Kα_1_ and Kα_2_ emission lines. In layered PbI_2_ materials, changes in the line shape of the (001) reflection have been associated with variations in stacking coherence and crystallite size along the *c*-axis.^9^ In the present analysis, the fitted variables were the Kα_1_ position of each doublet, the Gaussian and Lorentzian widths, and the integrated intensity of each doublet. The same Thompson-Cox-Hastings profile function, Kα doublet treatment, and linear-background model were applied to all samples to enable direct comparison of the resolved component areas. The resulting basal-spacing parameters, relative fitted areas, fitted widths, total full-width-half-maximum (fwhm) values, and apparent Scherrer coherence lengths are summarized in Table 2.

**Table 2:** Parameters Obtained from the Whole-Pattern Profile-Matching Analysis of the PbI_2_ (001) Basal Reflection

sample	component	2θ (°)	c (Å)	relative area (%)	*H_G_* (°)	*H_L_* (°)	*H*_tot_ (°)	*D*_app_ (nm)
s00	2H-1	12.6508	6.9916	90.47	0.59	0.06	0.62	12.84
	2H-2	13.0317	6.7881	9.53	0.34	0.06	0.37	21.42
s25	2H-1	12.7001	6.9646	96.35	0.65	0.01	0.66	12.20
	2H-2	13.0980	6.7539	3.65	0.24	0.01	0.25	32.59
s45	2H-1	12.6632	6.9848	100.00	0.62	0.00	0.62	12.89
s65	2H-1	12.7146	6.9567	92.61	0.69	0.00	0.69	11.58
	2H-2	13.0729	6.7668	7.39	0.35	0.00	0.35	22.85
s100	2H-1	12.6620	6.9854	93.43	0.66	0.00	0.66	12.11
	2H-2	13.1198	6.7427	6.57	0.30	0.00	0.30	26.66

For s00, the fitted envelope resolves into two 2H-like basal-spacing components within the layered PbI_2_ framework. The dominant component appears at 12.65°, corresponding to *c* = 6.99 Å, while the weaker component appears at 13.03°, corresponding to *c* = 6.79 Å. Their relative fitted areas are 90.47% and 9.53%, respectively. For s25, the dominant component shifts to 12.70° (*c* = 6.96 Å), while the secondary component shifts to 13.10° (*c* = 6.75 Å). The corresponding populations are 96.35% and 3.65%. These shifts indicate that both resolved basal-spacing environments contract relative to s00 after low-level MXene incorporation.

For s45, the basal envelope is adequately described by a single resolved 2H-like component at 12.66°, corresponding to *c* = 6.98 Å. Compared with s25, this shift to lower 2θ indicates a partial relaxation or expansion of the average basal spacing after the initial contraction induced at low MXene incorporation. The absence of a resolved secondary component suggests that, within the adopted fitting model, a second contracted basal-spacing contribution cannot be robustly distinguished at this intermediate MXene content. The fitted profile for s45 is dominated by a Gaussian contribution, and the Lorentzian component decreases to zero within the fitting uncertainty. This result should not be interpreted as direct proof that strain alone controls the broadening. Rather, it indicates that the phenomenological line shape does not require a resolvable Lorentzian contribution under the adopted fitting constraints.

For s65, the main component shifts to 12.71° (*c* = 6.96 Å), while the secondary component appears at 13.07° (*c* = 6.77 Å). Their relative fitted areas are 92.61% and 7.39%, respectively. For s100, the dominant component returns to 12.66° (*c* = 6.99 Å), whereas the secondary component shifts to 13.12° (*c* = 6.74 Å), with relative populations of 93.43% and 6.57%. These results show that the dominant 2H-like basal-spacing environment remains close to *c* ≈ 6.96–6.99 Å across the series, whereas the secondary environment remains consistently contracted, with *c* ≈ 6.74–6.79 Å.

To complement the basal-envelope analysis, we estimated an apparent Scherrer coherence length from the total fitted fwhm of each component. The total Voigt-like fwhm was approximated as

1 $$H_{\mathrm{tot}}=0.5346H_{L}+\sqrt{H_{G}^{2}+0.2166H_{L}^{2}}$$

Where *H*_*G*_ and *H*_*L*_ are the Gaussian and Lorentzian fwhm values, respectively. The apparent coherence length was then calculated using

2 $$D_{\mathrm{app}}=\frac{K\lambda }{\beta_{\mathrm{tot}}\cos\theta }$$

Where *K* = 0.9, λ = 1.5406 Å, *β*_tot_ is the total fwhm in radians, and θ is the Bragg angle. Because the films are highly textured and the analysis is based on a single basal envelope, *D*_app_ is interpreted as an apparent [001] coherence-length parameter rather than an absolute crystallite size.

The apparent Scherrer coherence length of the dominant component remains relatively stable, ranging from 11.58 to 12.89 nm. This narrow range indicates that MXene incorporation does not drastically reconstruct or collapse the average layered PbI_2_ framework. However, the lowest *D*_app_ value is observed for s65, where the basal-envelope decomposition also shows a stronger contribution from the contracted secondary component. This trend coincides with the second morphological transition observed at higher MXene loading, where the crystallites become more clustered and locally heterogeneous, suggesting that higher MXene loading perturbs vertical stacking coherence. In contrast, s45 shows only one resolved 2H-like basal-spacing component, with *D*_app_ = 12.89 nm, indicating partial basal-spacing relaxation relative to the MXene-containing samples with contracted components and a more uniform average stacking environment within the adopted model. This behavior is particularly relevant because it occurs at intermediate MXene incorporation, where the structural perturbation appears less distributed across distinct basal-spacing populations.

Taken together, the XRD results suggest that MXene incorporation preserves the layered 2H–PbI_2_ framework while producing composition-dependent changes in the basal-envelope line shape. Within the adopted phenomenological model, these changes are consistent with variations in the relative fitted-contribution of two average basal-spacing components. The close inspection of the (001) envelope indicates changes in vertical stacking coherence and interlayer spacing rather than a bulk structural transformation.

Raman spectroscopy was used to probe local lattice dynamics, stacking-related disorder, and interfacial vibrational perturbations in the PbI_2_–MXene composite films. This analysis complements the XRD basal-envelope decomposition because Raman scattering is sensitive to local symmetry, polarizability changes, and short-range stacking perturbations that may not appear as a new long-range crystalline phase. All spectra were normalized to the total integrated intensity within the 40–260 cm^–1^ spectral window to enable direct comparison of relative component contributions across the series, with the exception of the pristine MXene spectrum that required × 10 rescaling to better appreciate its spectral features. The measurements were acquired under identical instrumental conditions (laser power, integration time, and objective), minimizing thermal and instrumental contributions to relative frequency shifts and intensity variations.

Across the complete compositional series, the films retain the characteristic low-frequency Raman fingerprint of layered 2H–PbI_2_, as shown in Figure 5. Because XRD confirms preservation of the hexagonal 2H–PbI_2_ framework, we assign the Raman features using the D_3_d symmetry representation of 2H–PbI_2_. In this framework, the zone-center vibrational representation is Γ = A_1g_ + 2A_2u_ + 2E_u_ + E_g_, where E_g_ and A_1g_ are the first-order Raman-active modes, whereas A_2u_ and E_u_ are polar infrared-active modes.^56^ Accordingly, the bands centered at 70.51 ± 0.70 and 93.95 ± 0.43 cm^–1^ are assigned to the first-order Raman-active Eg in-plane shear mode and A_1g_ out-of-plane symmetric I–Pb–I breathing vibration, respectively. The dominant broad component centered at 106.58 ± 0.75 cm^–1^ and the shoulder at 112.61 ± 0.67 cm^–1^ are assigned to disorder-/resonance-activated polar LO-region contributions, mainly E_u_(LO)/A_2u_(LO)-related. These assignments are consistent with reported PbI_2_ Raman bands near 73–74, 95, and 110 cm^–1^ in bulk crystals, thin films, nanostructures, and chemically deposited PbI_2_, where the same spectral region is sometimes described using labels such as E_2_^1^, A_1_^1^, A_2u_, or A_1_^2^.^57–61^

**Figure 5:**
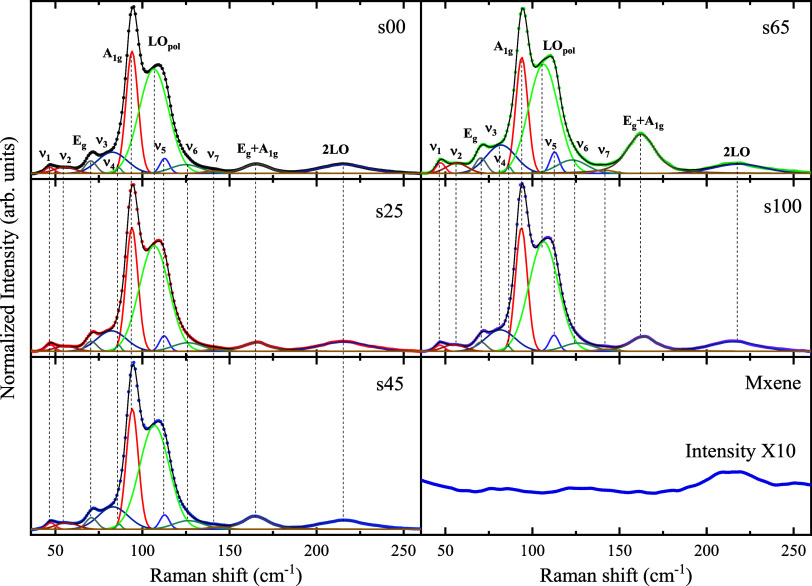
Raman spectra of PbI_2_–MXene films (s00–s100), together with fitted components and assignments defined in Table 3. The spectra retain the characteristic 2H–PbI_2_ fingerprint across all compositions, including the first-order E_g_ and A_1g_ modes, polar LO-region contributions, and higher-order E_g_+A_1g_ and 2LO bands.

The higher-frequency features are treated as second-order or combination bands rather than independent first-order Raman-active modes. Specifically, the band located at 164.47 ± 2.82 cm^–1^ is assigned to an E_g_+A_1g_ combination band, while the broad feature near 216.49 ± 2.43 cm^–1^ is assigned to a second-order LO-derived overtone, denoted here as 2LO [≈ 2 × A_2u_(LO)]. Some PbI_2_ studies also use labels such as 2E_1_^2^, 2E_2_^1^, 2E_1_^1^, or 2A_1_^2^ for these spectral regions.^57,59,60,62,63^ However, Table 3 uses E_g_+A_1g_ and 2LO to remain consistent with the 2H–PbI_2_ symmetry assignment and to avoid implying that these higher-frequency features are first-order irreducible representations.

**Table 3:** Raman Component Labels, Representative Band Positions, Vibrational Assignments, and Peak-Width Constraints Used for Fitting the PbI_2_–MXene Spectra

			peak width fwhm		
label	band position (cm^–1^)	vibrational mode identification	Gaussian (cm^*–*1^)	Lorentzian (cm^*–*1^)	reference
ν_1_	47.12 ± 0.90	Disorder-activated low-frequency acoustic/layer-breathing-related feature. Not a fundamental Γ-point Raman mode.	6.1 ± 1.1	0.0	^56,57,59,63^
ν_2_	55.81 ± 2.24	Disorder-/resonance-activated low-frequency polar feature, close to E_u_(TO)-like vibrations reported for 2H–PbI_2_.	14.5 ± 2.5	0.0	^56,57,63^
E_g_	70.51 ± 0.70	First-order Raman-active in-plane shear mode of 2H–PbI_2_. Reported as E_2_^1^ in some literature.	7.7 ± 1.2	0.0	^7,56–60,62,63^
ν_3_	82.34 ± 2.22	Weak E_g_-related shoulder or zone-folded/disorder-activated component. Not assigned as an independent first-order mode.	20.0 ± 3.5	0.0	^56,57,59^
ν_4_	86.40 ± 1.11	Weak disorder-/stacking-activated feature in the E_g_/A_1g_ spectral region. Not a fundamental Raman mode.	3.4 ± 0.9	0.0	^56,63^
A_1g_	93.95 ± 0.43	First-order Raman-active out-of-plane symmetric/breathing I–Pb–I vibration of 2H–PbI_2_. reported as A_1_^1^ in some literature. This signal may overlap spectrally with A_2u_(TO) -like polar contributions.	8.1 ± 1.0	0.0	^7,56–60,62,63^
LO_pol_	106.58 ± 0.75	Dominant disorder-/resonance-activated polar LO-region component, mainly E_u_(LO)/A_2u_(LO)-related. Reported as A_2u_ or A_1_^2^ in some literature.	20.0 ± 4.0	0.0	^7,56,57,59,60,62,63^
ν_5_	112.61 ± 0.67	High-frequency shoulder of the polar LO region, possibly A_2u_(LO)-like or mixed E_u_(LO)/A_2u_(LO)-related.	6.3 ± 1.0	0.0	^56,57,59,60,63^
ν_6_	125.31 ± 4.73	Weak disorder-activated high-frequency polar LO-related feature, close to computed A_2_u(LO) values.	20.0 ± 3.0	0.0	^56^
ν_7_	141.88 ± 4.17	Resonance-/disorder-activated optical combination feature. A related feature near 136 cm^–1^ has been reported in spin-coated PbI_2_ thin films.	13.0 ± 2.5	0.0	^58^
E_g_+A_1g_	164.47 ± 2.82	Combination E_g_+A_1g_ band. Reported as 2E_1_^2^ or 2E_2_^1^ in some literature, but not used here as the main assignment.	11.7 ± 1.9	10.0 ± 2.0	^57,59,60,62,63^
2LO	216.49 ± 2.43	Second-order LO-derived overtone, 2LO-type [≈ 2 × A_2u_(LO)]. Often denoted 2A_2_u in PbI_2_ literature. Assigned as 2E_1_^1^ or 2A_1_^2^ in some literature, but are treated here only as historical alternatives.	36.5 ± 4.2	0.0	^57–60,62,63^

The persistence of the E_g_, A_1g_, LO-region, E_g_+A_1g_, and 2LO features across all compositions indicates that Ti_3_C_2_T_*x*_ incorporation preserves the fundamental I–Pb–I layered motif and does not induce a bulk structural transformation. This conclusion agrees with the XRD results, which show retention of the hexagonal PbI_2_ phase across the series. At the same time, Raman spectroscopy probes local vibrational environments that average diffraction may not fully resolve.^61,64^ The weaker components denoted as ν_1_–ν_7_ in Table 3, located at approximately 47.12 ± 0.90, 55.81 ± 2.24, 82.34 ± 2.22, 86.40 ± 1.11, 112.61 ± 0.67, 125.31 ± 4.73, and 141.88 ± 4.17 cm^–1^, are assigned to disorder-activated, polar-mode-related, zone-folded, or resonance-enhanced contributions. Their presence supports local symmetry relaxation and stacking-related disorder within the layered PbI_2_ lattice,^57–60,64^ rather than the formation of a new crystalline phase. This interpretation agrees with the asymmetric PbI_2_ (001) basal envelope observed by XRD, which was consistently described using more than one 2H-like stacking contribution in most compositions.

For most compositions, the fitted Raman peak positions remain nearly invariant, with shifts generally limited to approximately 1–2 cm^–1^ (Figure 6, right panel). This limited variation is particularly evident for the first-order E_g_ and A_1g_ modes, indicating that the dominant Pb–I vibrational framework of 2H–PbI_2_ remains largely preserved after MXene incorporation. This behavior is consistent with the XRD analysis, where the main 2H-like basal-spacing component remains close to *c* ≈ 6.96–6.99 Å across the series, and with the absence of new Pb-containing crystalline phases. Therefore, the Raman spectra support a model in which MXene incorporation modifies local stacking and interfacial environments without producing a global reconstruction of the PbI_2_ lattice.^65^

**Figure 6:**
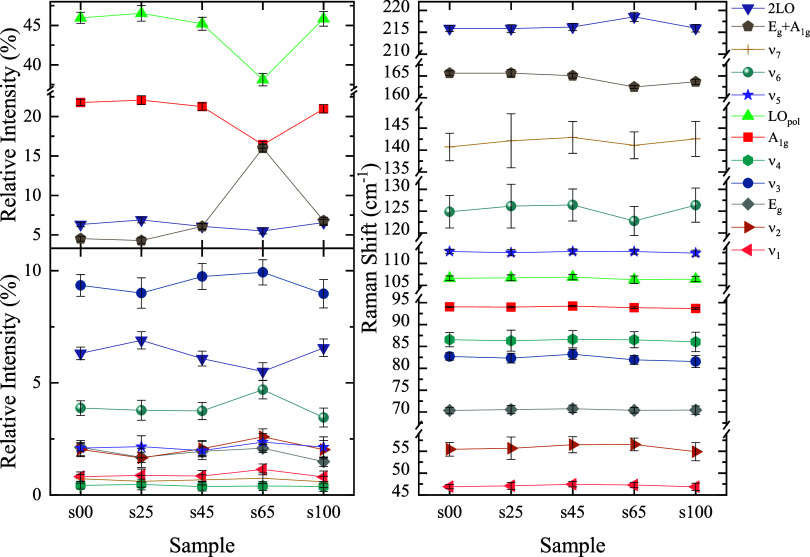
(Left panel) Relative intensity contributions of fitted Raman components as a function of sample composition (s00–s100). (Right panel) Raman peak positions of the fitted components across the same series, showing minimal frequency shifts but composition-dependent intensity redistribution.

A more pronounced and mode-selective vibrational response is observed at s65. This composition also shows the lowest apparent [001] coherence length for the dominant XRD component and a re-emergent contracted 2H-like basal-spacing contribution, while SEM shows a second morphological transition characterized by shorter, broader, and less sharply defined platelet-like features with local clustering. At s65, the 2LO band shifts from 215.87 ± 0.67 cm^–1^ in s00 to 218.53 ± 1.04 cm^–1^, corresponding to a blue shift of +2.66 cm^–1^. In contrast, the E_g_+A_1g_ combination band shifts from 165.61 ± 0.56 cm^–1^ to 162.46 ± 0.35 cm^–1^, while the disorder-/polar-LO-related ν_6_ component shifts from 124.85 ± 3.70 cm^–1^ to 122.75 ± 3.34 cm^–1^. In particular, the E_g_+A_1g_ displacement clearly exceeds its fitting uncertainty, whereas the ν6 trend is consistent with red shifting but is less precisely resolved because this weak, broad component carries a larger positional uncertainty.

The opposite signs of these frequency shifts indicate that the PbI_2_ vibrational response is not governed by a uniform lattice expansion or contraction. Instead, the second-order and polar-LO-related components respond selectively to the distribution of local stacking environments resolved by XRD. The blue shift of the 2LO band suggests local stiffening of LO-derived vibrational contributions or a change in the weighting of LO-related overtone processes, which may arise from constrained lattice motion near Ti_3_C_2_T_*x*_ nanosheets, locally contracted basal-spacing environments, or modified interlayer coupling. Conversely, the red shifts of the E_g_+A_1g_ and ν_6_ features suggest local phonon softening associated with stacking disorder, microstrain heterogeneity, or relaxation of specific interlayer configurations. Because Ti_3_C_2_T_*x*_ surfaces contain chemically active −O, −OH, and −F terminations, interfacial electrostatic screening and local charge redistribution may also modify selected Pb–I vibrational force constants. Thus, the Raman response is best interpreted as mode-selective phonon renormalization arising from heterogeneous stacking and interfacial coupling, rather than as evidence of a new crystalline phase.

The relative intensity contributions of the fitted Raman components also evolve with MXene loading (Figure 6, left panel). The strongest redistribution occurs at s65, where the E_g_+A_1g_ combination band increases markedly while the dominant LO_pol_ and A_1g_ contributions decrease relative to the other compositions. This behavior should not be interpreted simply as a change in the average lattice parameter. Instead, it is more consistent with a redistribution among local stacking environments, changes in Raman scattering efficiency, local polarizability derivatives, crystallite orientation, and symmetry-dependent selection rules. This interpretation agrees with the XRD basal-envelope analysis, which was used to track changes in the relative fitted contributions of 2H-like basal-spacing components, and with the SEM evidence of a second morphological transition at s65. In textured layered films, such intensity variations can arise from changes in stacking coherence, planar-fault density, local symmetry breaking, and orientation distributions.^61^

The possible contribution of Ti_3_C_2_T_*x*_ to the low-frequency spectra must also be considered. Pristine MXene can exhibit Raman features in the approximately 200–220 cm^–1^ region associated with Ti–C vibrations and surface-termination-dependent modes.^66,67^ However, the composite spectra remain dominated by PbI_2_-related vibrational features within the 40–260 cm^–1^ window. This dominance is consistent with the relatively low MXene fraction, attenuation or overlap of MXene contributions within the PbI_2_ matrix, and the limited Raman visibility expected for dispersed or structurally disordered MXene domains. Therefore, we attribute the observed shifts and intensity redistribution primarily to changes in the PbI_2_ local stacking and interfacial vibrational environment induced by MXene incorporation, rather than to the emergence of an independent MXene Raman signature.

### 3.4 Chemical State Analysis

XPS analysis was performed to evaluate the chemical states and possible electronic interactions in the PbI_2_–MXene films (Figure 7). The Pb 4f spectra of all samples exhibit the characteristic doublet associated with Pb–I bonding, with the Pb 4f_7/2_ component centered between approximately ∼138.30 ± 0.02 and 138.50 ± 0.03 eV, consistent with reported values for PbI_2_.^7,58,68^ A minor lower-binding-energy component near 136.60 ± 0.25 eV is assigned to metallic Pb^0^,^7,58,68,69^ which may arise from partial surface reduction under X-ray exposure or vacuum conditions.^68^ A weak feature near 139.20 ± 0.30 eV is attributed to residual Pb­(NO_3_)_2_ ^68,69^ species from the precursor solution.

**Figure 7:**
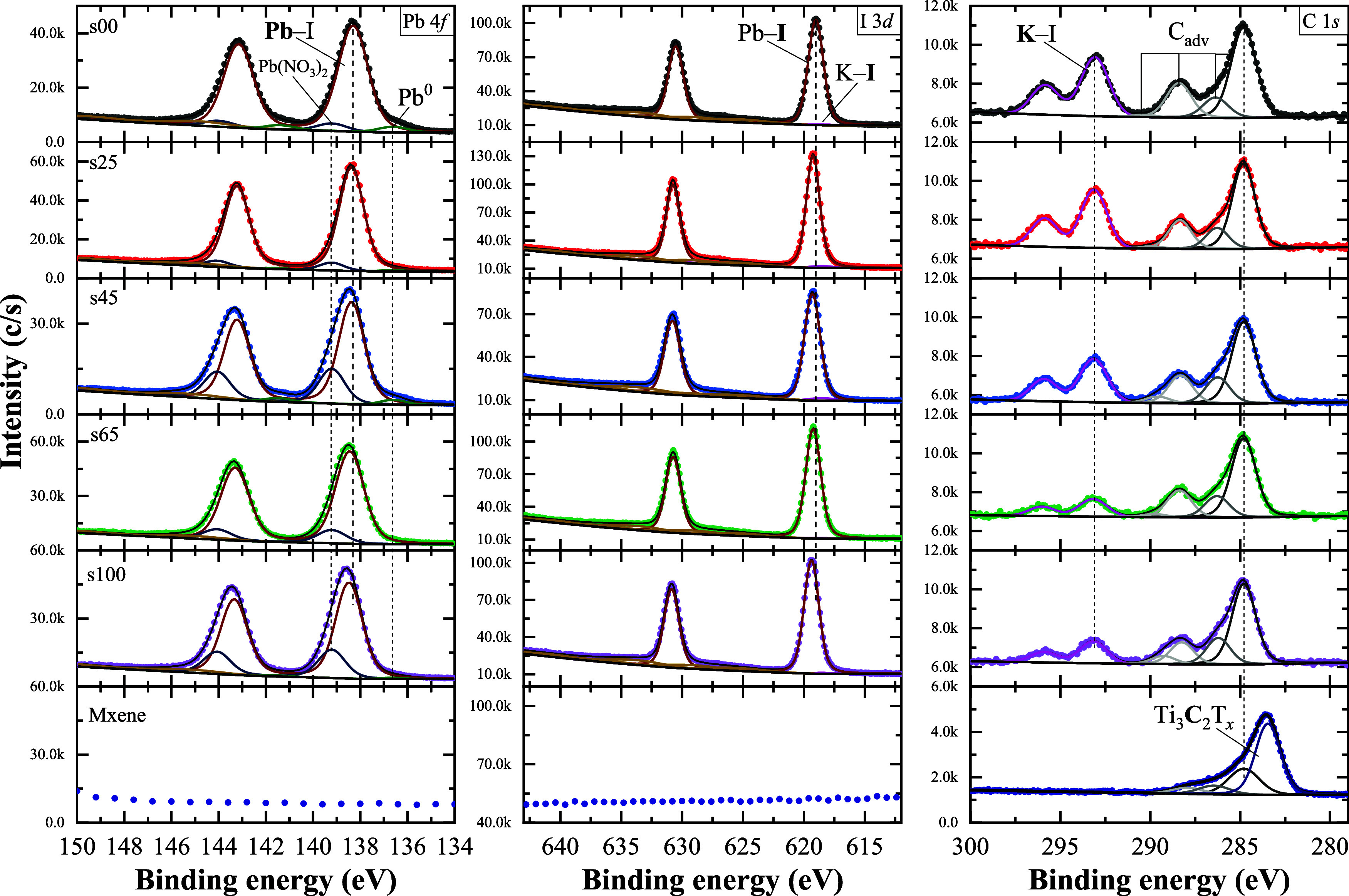
High-resolution XPS spectra of PbI_2_–MXene films (s00–s100) and Ti_3_C_2_T_*x*_ reference: (left) Pb 4f region showing Pb–I, Pb^0^, and residual Pb­(NO_3_)_2_ components; (center) I 3d region showing Pb–I and minor K–I contributions; (right) C 1*s* region with fitted components, including adventitious carbon used for charge calibration and metal carbide contribution in the MXene reference.

With increasing MXene incorporation, the Pb–I component exhibits a gradual shift toward higher binding energy relative to pristine PbI_2_. The total shift between s00 and s100 is approximately 0.3–0.4 eV, exceeding the experimental step size of 0.12 eV and remaining larger than the estimated fitting uncertainty. The I 3d region shows the expected doublet corresponding to iodide bound to Pb, with the I 3d_5/2_ peak located at 619.01 ± 0.01 eV in s00. A minor component near 618.50 ± 0.25 eV is attributed to K–I species derived from precursor residues. Similar to the Pb 4f region, the I–Pb component shifts toward higher binding energy with increasing MXene content, reaching 619.36 ± 0.03 eV in s100.

Importantly, the components associated with residual precursor species (Pb­(NO_3_)_2_ and K–I) remain at constant binding energy across the series within experimental uncertainty. This selective shift of the Pb–I related components indicates that the observed evolution is not associated with global spectral displacement. This result further supports that the statistically significant Pb–I core-level shifts arise from changes in the Pb–I chemical environment rather than from a uniform calibration or charging artifact.

Binding energies were referenced to the adventitious carbon peak at 284.8 ± 0.05 eV. To further evaluate the possibility of uniform surface-charging effects, we analyzed the binding-energy difference between the Pb 4f_7/2_ and I 3d_5/2_ peaks (Figure 8). Under uniform electrostatic charging, this separation should remain constant because both core levels would shift by the same energy offset. Instead, the Pb–I binding energy difference shows a statistically significant and nonmonotonic dependence on MXene loading. The pristine s00 film exhibits the lowest separation, whereas all MXene-containing films show larger Pb 4f–I 3d separations within the 95% confidence intervals. The separation increases from s25 to s45, decreases significantly at s65, and increases again at s100 to values comparable to the higher-separation MXene-containing samples. This behavior indicates that the spectral evolution cannot be fully explained by uniform charging and instead reflects changes in the local electrostatic and chemical environment of the Pb–I framework. Because the absolute shifts remain small and the Pb^2+^/I^–^-related spectral assignments are preserved, these changes do not indicate a change in oxidation state. Rather, they suggest that Ti_3_C_2_T_*x*_ incorporation modifies the local electronic distribution and core-level potential around Pb–I bonding environments.

**Figure 8:**
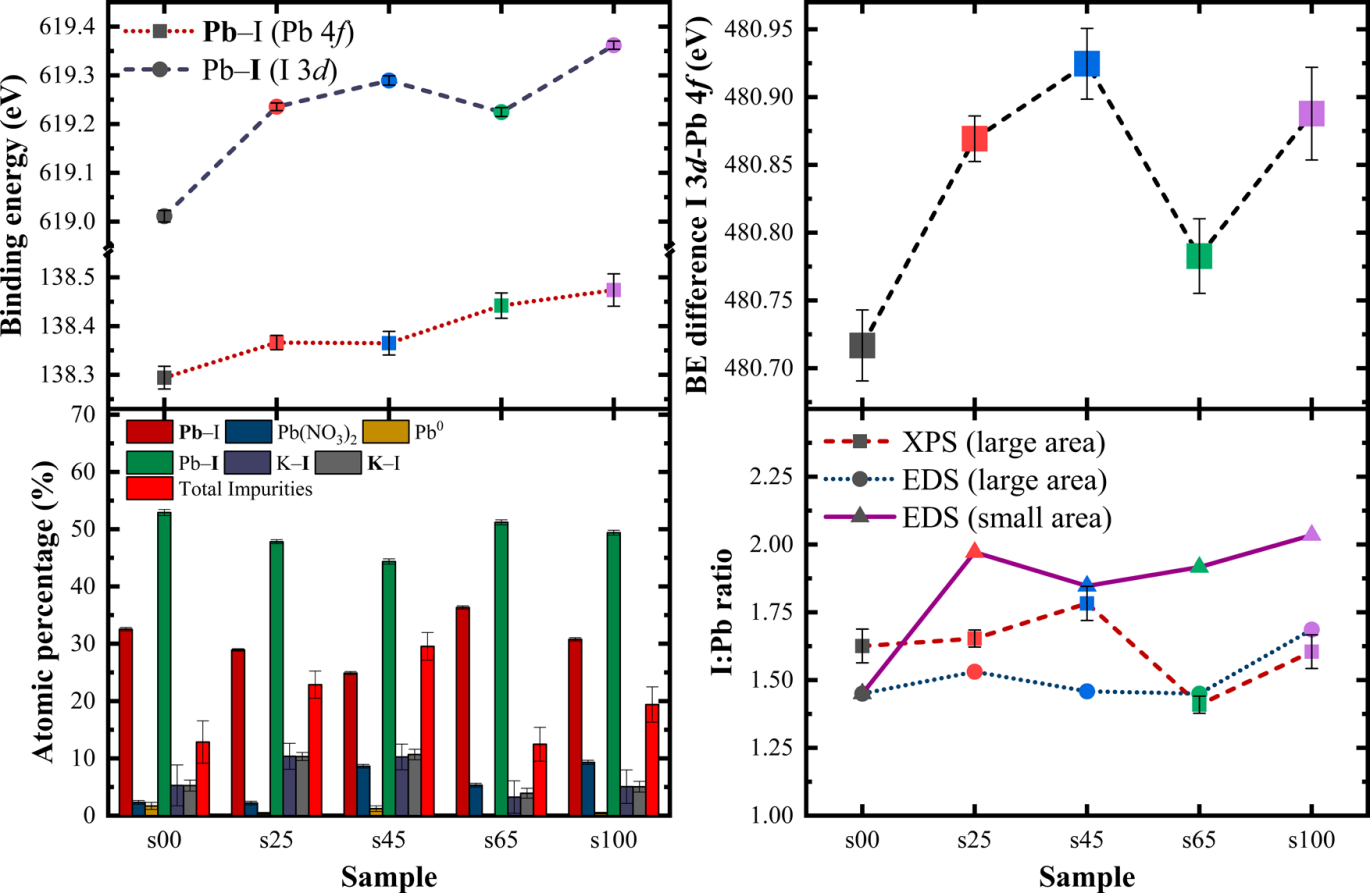
Summary of XPS quantitative analysis: (top left) Binding energy evolution of Pb 4f and I 3d core levels; (top right) Binding energy difference between Pb 4f and I 3d as a function of composition, with error bars representing the estimated uncertainty; (bottom left) Atomic percentage of fitted chemical species; (bottom right) I:Pb atomic ratio determined by XPS, large-area EDS, and small-area EDS collected from bright Ti-rich clustered regions.

The C 1s spectra show the dominant adventitious carbon component at 284.80 eV together with higher-binding-energy contributions attributed to oxygenated carbon species and the K 2p spectra related to residual KI precursor. In the Ti_3_C_2_T_*x*_ reference spectrum, a Ti–C carbide component is clearly observed at lower binding energy. In contrast, the Ti–C contribution is not distinctly resolved in the composite films, consistent with the relatively low MXene concentration and partial attenuation of MXene-related signals beneath a PbI_2_ overlayer.

Quantitative analysis reveals that the dominant chemical environment across all compositions corresponds to Pb–I bonding. The combined atomic fraction of Pb–I and I–Pb components accounts for approximately 75–85% of the fitted near-surface chemical species, indicating that under the present processing conditions, the Pb–I coordination remains the dominant chemical environment after MXene incorporation. The remaining fraction is attributed to minor precursor-derived species, including residual nitrate-related Pb components, metallic Pb contributions, and trace potassium-related species at low concentration.

The Pb–I bonded fraction reported here corresponds to the total intensity-corrected fitted contribution associated with Pb–I bonding environments, calculated from both the Pb 4f and I 3d core-level regions. Because XPS is surface sensitive, with an estimated sampling depth of approximately 12 nm, this value reflects the near-surface chemistry of the thin films rather than an absolute bulk conversion yield. Nevertheless, the analysis provides robust spectroscopic evidence that Pb–I bonding remains the dominant chemical environment across the full composition series, because the quantification considers both Pb 4f and I 3d contributions rather than relying only on the fraction of Pb atoms detected in the Pb 4f spectra. Such chemically resolved quantification of bonded Pb–I environments is not commonly reported for solution-processed PbI_2_ thin films, where phase formation is typically inferred from diffraction data alone. Therefore, the present analysis provides a direct spectroscopic evaluation of the near-surface chemical conversion toward Pb–I coordination at the atomic level, complementing the XRD and Raman results that show preservation of the 2H–PbI_2_ framework.

The I:Pb atomic ratio determined by XPS remains slightly substoichiometric relative to the ideal 2:1 ratio but remains consistent across all compositions. Small deviations from ideal stoichiometry are frequently observed in solution-processed PbI_2_ thin films, particularly at the surface, where halogen loss or surface chemical modification may occur.^7,58^ The large-area EDS ratios follow the same substoichiometric tendency, indicating that the iodine deficiency is not limited to the near-surface region probed by XPS. In contrast, the small-area EDS analysis performed on bright Ti-rich clustered regions gives I:Pb ratios closer to the ideal PbI_2_ value, particularly in the higher MXene-incorporation samples. This comparison suggests that the Ti-rich regions identified by SEM-EDS are associated with more PbI_2_-like local stoichiometry, supporting their role as preferential sites for Pb–I accumulation or local crystallite organization. Overall, the XPS results indicate that MXene incorporation induces subtle but systematic modifications of the Pb–I core-level binding energies while preserving the fundamental chemical state of PbI_2_.

### 3.5 Optical Properties

The optical response of the PbI_2_–MXene films was evaluated through transmittance and reflectance measurements, from which the absorption coefficient (α) was calculated using the measured film thickness values (Figure 9). All samples exhibit strong absorption in the visible region, consistent with the direct optical bandgap (∼2.3–2.5 eV) typically reported for PbI_2_ thin films.^7,58^ The absorption edge remains sharp across the entire composition range, indicating the preservation of the 2H–PbI_2_ optical response after MXene incorporation.

**Figure 9:**
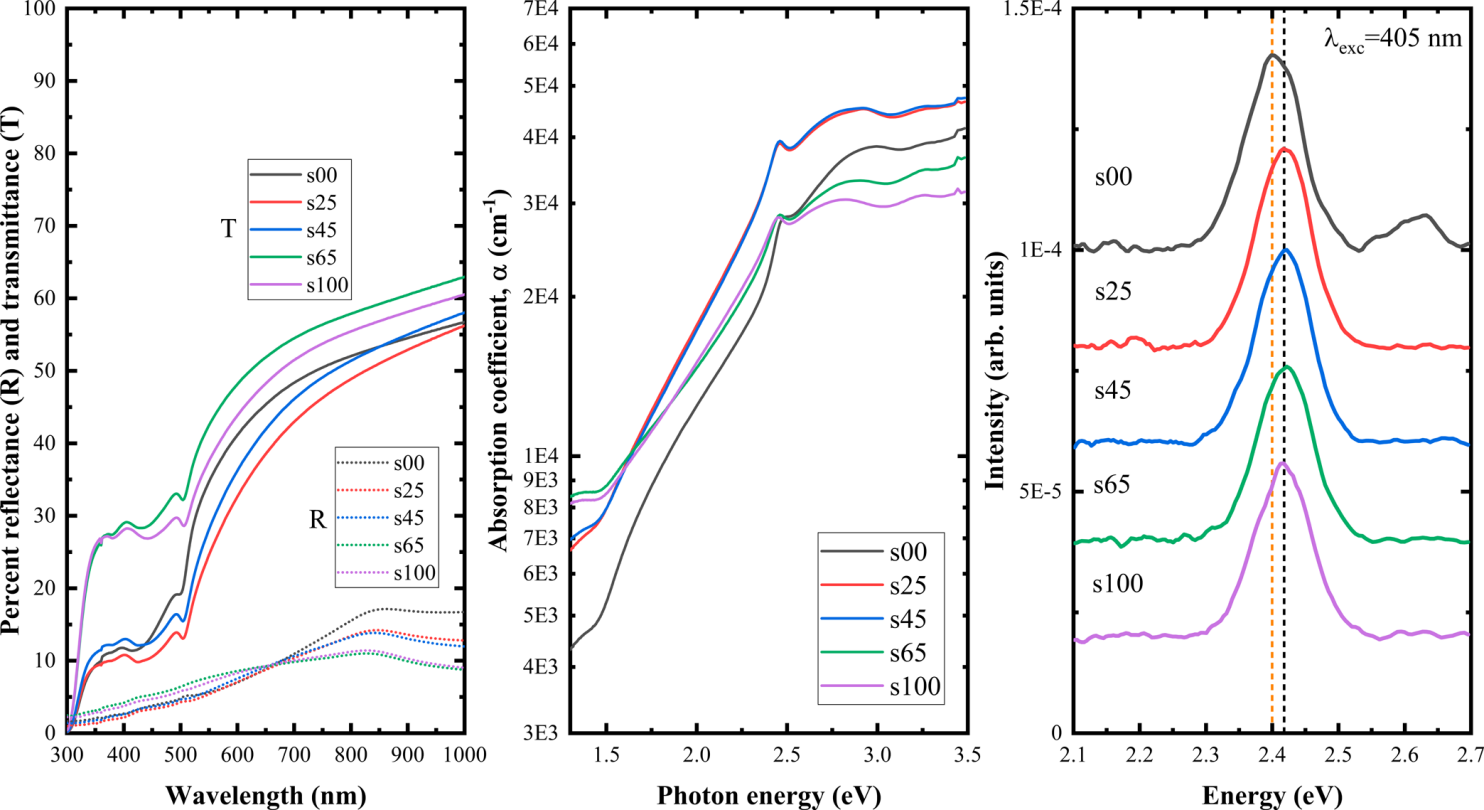
Optical characterization of PbI_2_–MXene films (s00–s100). (Left) Transmittance (T) and reflectance (R) spectra. (Center) Absorption coefficient (α) as a function of photon energy, showing preserved PbI_2_–related absorption edge and a slight near-edge excitonic shift with MXene incorporation. (Right) Photoluminescence spectra acquired under 405 nm excitation, showing progressive emission quenching and a small blue shift of the emission maximum in the MXene-containing films.

Although the absorption edge position remains largely unchanged, the near-edge absorption feature shows a small blue shift after MXene incorporation. The limited magnitude of this shift suggests a modest modification of the local electronic environment rather than a substantial change in the intrinsic band structure of PbI_2_. Therefore, the optical response is more consistent with subtle interfacial or local-structure perturbation than with the formation of a new optical phase.

The magnitude of the absorption coefficient varies with composition, with s25 and s45 showing the highest absorption in the near-edge and high-energy regions, whereas s65 and s100 show lower α values. Because profilometry shows that the average film thickness remains nearly constant across the series, these changes cannot be assigned to a systematic thickness variation. Instead, the nonmonotonic evolution of α more likely reflects differences in local morphology, surface roughness, scattering, and MXene-related optical attenuation. The preservation of the absorption edge despite these intensity variations indicates that MXene incorporation modifies optical losses and local electronic environments without substantially changing the intrinsic PbI_2_ band-edge structure.

Photoluminescence (PL) spectra acquired under 405 nm excitation (Figure 9, right panel) show a progressive reduction in emission intensity as the MXene content increases. The pristine film, s00, exhibits the strongest emission, whereas s100 shows the most pronounced quenching. In addition to intensity reduction, the PL maximum shifts slightly from approximately 2.40 eV in s00 to approximately 2.42 eV in MXene-containing films. This small blue shift parallels the near-edge shift observed in the absorption spectra and supports a modest modification of the local electronic environment after MXene incorporation. Because time-resolved PL was not measured in the present study, we cannot assign a specific lifetime component or carrier-transfer rate. Nevertheless, the steady-state PL quenching is consistent with previous reports on Ti_3_C_2_T_*x*_-containing halide systems, where MXene incorporation shortens PL lifetimes and promotes interfacial carrier extraction.^4,51,70^ A similar role of conductive two-dimensional components in promoting charge separation or carrier redistribution has been reported in broader MXene-based hybrid systems under illumination, although the present PbI_2_-MXene films are evaluated here through steady-state PL and J-V/photoresponse rather than time-resolved carrier dynamics.^32^

The systematic PL quenching suggests that MXene incorporation enhances nonradiative pathways and/or facilitates interfacial carrier extraction. Under 405 nm excitation, PbI_2_ absorbs above its band edge and generates excited carriers or excitonic states that can recombine radiatively near the observed emission energy. In the composite films, Ti_3_C_2_T_*x*_-derived regions introduce two plausible classes of quenching sites. First, the conductive electronic states of Ti_3_C_2_T_*x*_ can act as electron-accepting or charge-redistributing sites for photoexcited PbI_2_. Second, the surface terminations of MXene and the Pb–I/MXene contact regions can introduce interfacial trap states that favor nonradiative recombination or reversible trapping/detrapping of photogenerated carriers. Bodunov and Simões Gamboa showed, in a different semiconductor nanostructure context, that interfaces and trap states can strongly affect PL decay kinetics because trapping and detrapping processes condition the fate of photogenerated carriers.^70^ By analogy, the PL quenching observed here can be rationalized by a combination of electron extraction into MXene-derived conductive states, trap-assisted nonradiative recombination at PbI_2_/MXene interfaces, and possible hole trapping or redistribution at iodine-related or surface-termination-related sites. Because time-resolved PL was not measured, we do not assign one dominant carrier-transfer pathway. Instead, we interpret the PL decrease as evidence of enhanced interfacial nonradiative deactivation consistent with the statistically significant Pb 4f–I 3d binding-energy separation observed by XPS, which indicates that MXene incorporation modifies the local electrostatic and chemical environment around Pb–I bonding sites.

Although s65 exhibited the strongest structural and vibrational perturbations in the XRD and Raman analyses, it does not display the strongest PL quenching. This decoupling indicates that the suppression of radiative recombination is not governed only by stacking-related disorder or basal-envelope modification. Instead, the PL response likely depends on the combined effect of local PbI_2_ structure, MXene spatial distribution, Ti-rich clustered regions, and the density of interfacial quenching sites. At higher MXene content, the larger population of Ti_3_C_2_T_*x*_-derived regions can increase the probability of carrier trapping, interfacial energy dissipation, or charge redistribution, even when the structural perturbation is not maximal.

### 3.6 Photoelectrical Response

The current density–voltage (*J*–*V*) characteristics of the PbI_2_–MXene films were measured under dark and illuminated conditions and presented in Figure 10. All measurements were performed under ambient conditions. The *J*–*V* curves display nearly linear behavior over the investigated voltage range, indicating predominantly ohmic-like transport under the present measurement geometry. Because the profilometry analysis shows nearly constant average film thickness across the series, the observed changes in current density and resistivity cannot be attributed to systematic thickness variation. Instead, they mainly reflect composition-dependent changes in the photoelectrical response associated with MXene incorporation.

**Figure 10:**
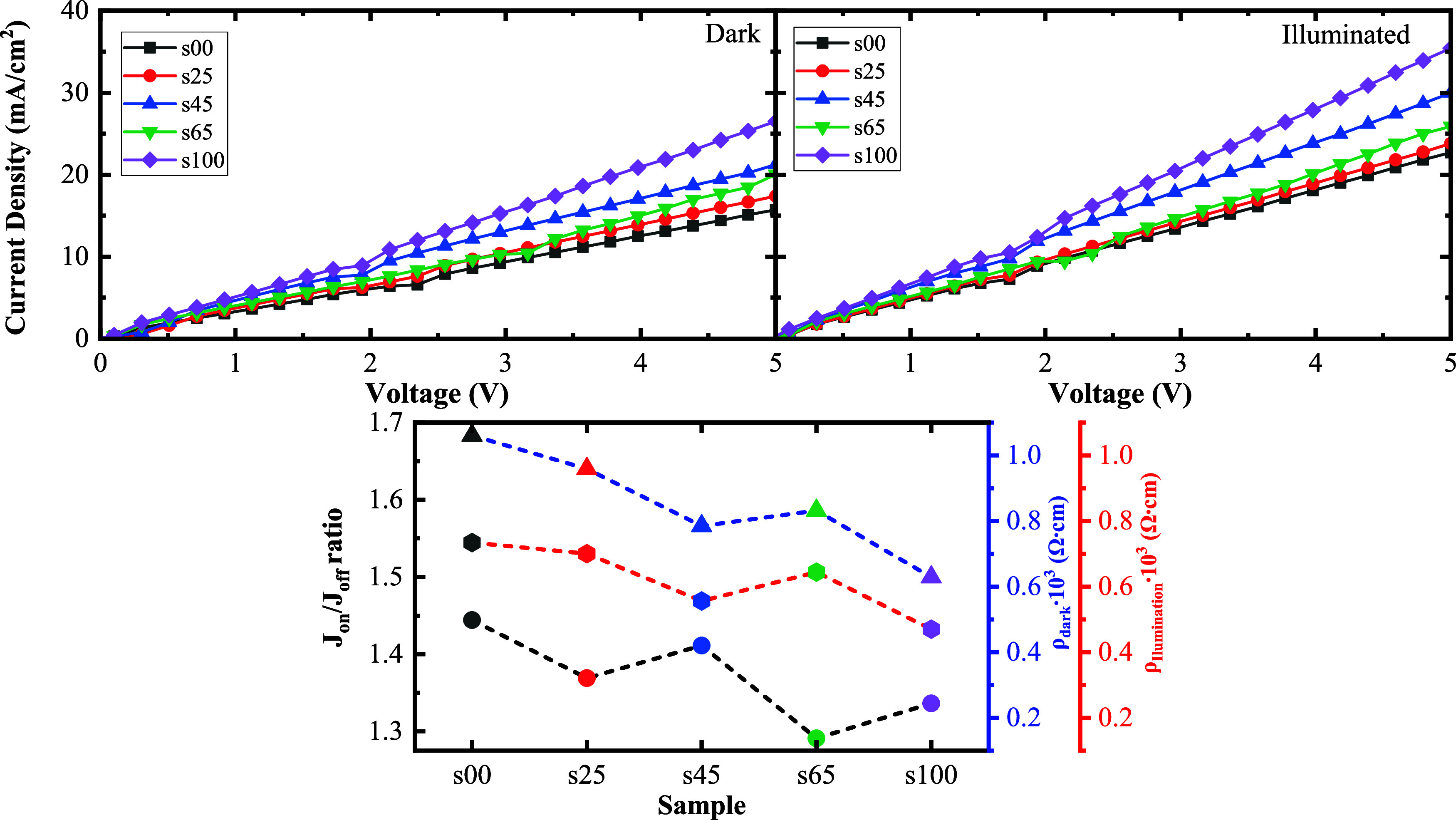
*J*–*V* characteristics of PbI_2_–MXene films (s00–s100) measured under dark conditions (Top left panel) and under illumination (Top right panel). Bottom panel shows the photoresponse metrics of PbI_2_–MXene films: J_on_/J_off_ ratio and resistivity under dark (ρ_dark_) and illuminated (ρ_illumination_) conditions.

Under dark conditions (Figure 10, top left panel), the current density increases with MXene incorporation. At 5 V, current density rises from 15.7 mA/cm^2^ to 26.35 mA/cm^2^, with the highest value observed for s100. This trend indicates that Ti_3_C_2_T_*x*_ incorporation increases the measured current density under the present measurement geometry. However, the evolution is not strictly monotonic, which suggests that conductivity does not depend only on nominal MXene loading. Rather, it also depends on the spatial distribution, connectivity, and local aggregation of Ti_3_C_2_T_*x*_-derived regions within the film.

This behavior is consistent with the SEM-EDS results, which show Ti-rich clustered regions in the MXene-containing films. At low and intermediate MXene loading, conductive Ti_3_C_2_T_*x*_-derived regions may remain partially separated within the PbI_2_ matrix, limiting continuous transport pathways. At higher loading, the increased density and proximity of Ti-rich regions can promote more effective connectivity, leading to enhanced current density.^71^

Under illumination (Figure 10, top right panel), all samples exhibit increased current density, compared to dark conditions, confirming their photoresponsive behavior. The largest absolute photocurrent is observed for s100, where the current density at 5 V increases from 26.35 mA/cm^2^ under dark conditions to 35.5 mA/cm^2^ under illumination, consistent with improved charge transport through MXene-assisted conductive pathways.^72^ This result agrees with the PL analysis, where stronger emission quenching at higher MXene content suggests enhanced nonradiative deactivation and/or interfacial carrier extraction. In this context, Ti_3_C_2_T_*x*_-derived regions may contribute to carrier redistribution after photoexcitation of PbI_2_, thereby increasing the measured current under illumination.

The photocurrent enhancement does not scale linearly with MXene loading. Although MXene incorporation improves electrical conductivity and can facilitate carrier extraction, high MXene content also increases the dark current. As a result, the relative contrast between illuminated and dark states does not necessarily improve at the same rate as the absolute photocurrent. This distinction explains why s100 shows the highest absolute current density but does not exhibit the highest J_on_/J_off_ ratio.

The bottom panel of Figure 10 summarizes the J_on_/J_off_ ratio together with resistivity values measured under dark (ρ_dark_) and illuminated (ρ_illumination_) conditions. For all compositions, the J_on_/J_off_ ratio remains greater than unity, confirming photoresponsive behavior. The pristine PbI_2_ film exhibits the highest relative photoresponse, whereas the MXene-containing samples show lower relative contrast because their dark conductivity increases.

Resistivity decreases under illumination for all samples, consistent with the contribution of photogenerated carriers. The magnitude of this resistivity modulation ranges from 1.06 × 10^3^ to 0.62 × 10^3^ Ω·cm, under dark conditions and from 0.73 × 10^3^ to 0.47 × 10^3^ Ω·cm under illumination. This response varies with composition, reflecting the combined influence of PbI_2_ photogeneration, MXene-associated carrier redistribution, and the heterogeneous distribution of Ti-rich regions across the composite films.

### 3.7 Proposed Thin Film Formation and MXene Incorporation Pathway

The combined profilometry, SEM-EDS, XRD, Raman, XPS, optical, and photoelectrical results support a growth-mediated interpretation for Ti_3_C_2_T_*x*_ incorporation into PbI_2_ thin films. The preservation of the 2H–PbI_2_ structure across all compositions, together with the absence of systematic lattice-parameter expansion, does not support bulk substitution of MXene into the PbI_2_ lattice or extensive postgrowth intercalation between PbI_2_ layers. Instead, the data support a formation pathway in which MXene-derived Ti-rich regions influence local Pb–I assembly, platelet development, stacking-related order, and the near-surface electrostatic environment during film growth.

#### 3.7.1 Precursor Solution Chemistry and Surface Activation

Under the water/ethanol solvent system employed here, Pb^2+^ and I^–^ exist as solvated species whose coordination environments differ substantially from those formed in strongly coordinating solvents such as DMF or DMSO. Literature on Pb­(NO_3_)_2_ solvation in mixed aqueous–alcohol systems indicates that Pb^2+^ exists predominantly as hydrated species, with partial ethanol substitution within its solvation shell, while nitrate acts primarily as a counterion in the mixed solvent environment (eq 3–4).^73–76^

3 $${\mathrm{Pb}}^{2+}+xH_{2}O⇄{\left[\mathrm{Pb}{\left(H_{2}O\right)}_{x}\right]}^{2+}$$

4 $${\left[\mathrm{Pb}{\left(H_{2}O\right)}_{x}\right]}^{2+}+n\mathrm{EtOH}⇄{\left[\mathrm{Pb}{\left(H_{2}O\right)}_{x-n}{\left(\mathrm{EtOH}\right)}_{n}\right]}^{2+}$$

Similarly, iodide in mixed solvents can participate in hydrogen-bond-mediated solvation and ion-pairing environments (eqs 5–7).^77–79^

5 $$\mathrm{KI}⇄K^{+}+I^{-}$$

6 $$I^{-}+m\mathrm{EtOH}⇄I^{-}\cdot \cdot \cdot {\left(\mathrm{EtOH}\right)}_{m}$$

7 $$I^{-}+xH_{2}O⇄{\left[I{\left(H_{2}O\right)}_{x}\right]}^{-}$$

During the first spin-coating step, deposition of the R-Pb solution onto UV–ozone treated glass promotes interaction between solvated Pb^2+^ species and surface silanol (Si–OH) groups. Surface complexation of Pb^2+^ at hydroxylated oxide interfaces is well documented in related systems and is therefore expected to generate localized adsorption sites that facilitate nucleation.^80,81^ Upon dispensing the R-I solution, iodide ions react with the surface-associated Pb^2+^ species, initiating PbI_2_ nucleation. Repetition of this sequential deposition cycle progressively builds layered PbI_2_ through surface-mediated growth. The final film preserves the 2H stacking sequence characteristic of layered PbI_2_, in which I–Pb–I layers are coupled through van der Waals interactions.^82^

#### 3.7.2 Growth-Mediated MXene Incorporation

When Ti_3_C_2_T_*x*_ is introduced into the R-I precursor, its surface terminations, including −O, −OH, and −F groups, can provide polar interfacial sites that may interact with solvated ionic species through electrostatic interactions, hydrogen bonding, and local ion association. This interpretation is consistent with broader MXene-composite literature showing that surface terminations, electrostatic self-assembly, interlayer/interfacial modification, and hybridization with conductive phases can regulate local structure and functional response in MXene-containing systems.^32–34^ Therefore, MXene-derived regions can locally modify the chemical environment encountered by iodide and Pb-containing species during the R-I deposition step. This proposed interfacial association is schematically represented in (eqs 8 and 9).^83–85^

8 $${\mathrm{Pb}}^{2+}+T_{3}C_{2}T_{x}⇄T_{3}C_{2}T_{x}-{\mathrm{Pb}}^{2+}$$

9 $${2I}^{-}+T_{3}C_{2}T_{x}-{\mathrm{Pb}}^{2+}⇄\mathrm{Pb}I_{2}\;\left(\mathrm{interface}\right)-T_{3}C_{2}T_{x}$$

Eqs 8 and 9 should be interpreted as schematic representations of interfacial association during film formation, not as evidence for discrete molecular complexes or fully resolved reaction intermediates. In this model, MXene-derived surfaces do not replace PbI_2_ lattice sites. Instead, they provide local contact regions where Pb- and I-containing species can accumulate and organize during the sequential deposition process.

Figure 11 summarizes this proposed growth-mediated pathway. The schematic compares the formation of pristine PbI_2_ films with the formation of PbI_2_:Ti_3_C_2_T_*x*_ composite films during sequential dynamic spin-coating. In the pristine route, the R-Pb deposition step promotes Pb-containing surface adsorption on hydroxylated glass, and the subsequent R-I deposition step promotes PbI_2_ formation. In the MXene-containing route, Ti_3_C_2_T_*x*_ is introduced through the R-I solution and may create MXene-derived Ti-rich contact regions near the growth front. These regions can interact with Pb- and I-containing species through surface-termination-mediated electrostatic interactions and hydrogen bonding, thereby modifying local Pb–I accumulation, crystallite organization, and stacking-related environments. Therefore, Figure 11 should be read as a correlation-supported formation model based on the experimental results discussed below, not as direct atomic-scale evidence of a fully resolved nucleation pathway.

**Figure 11:**
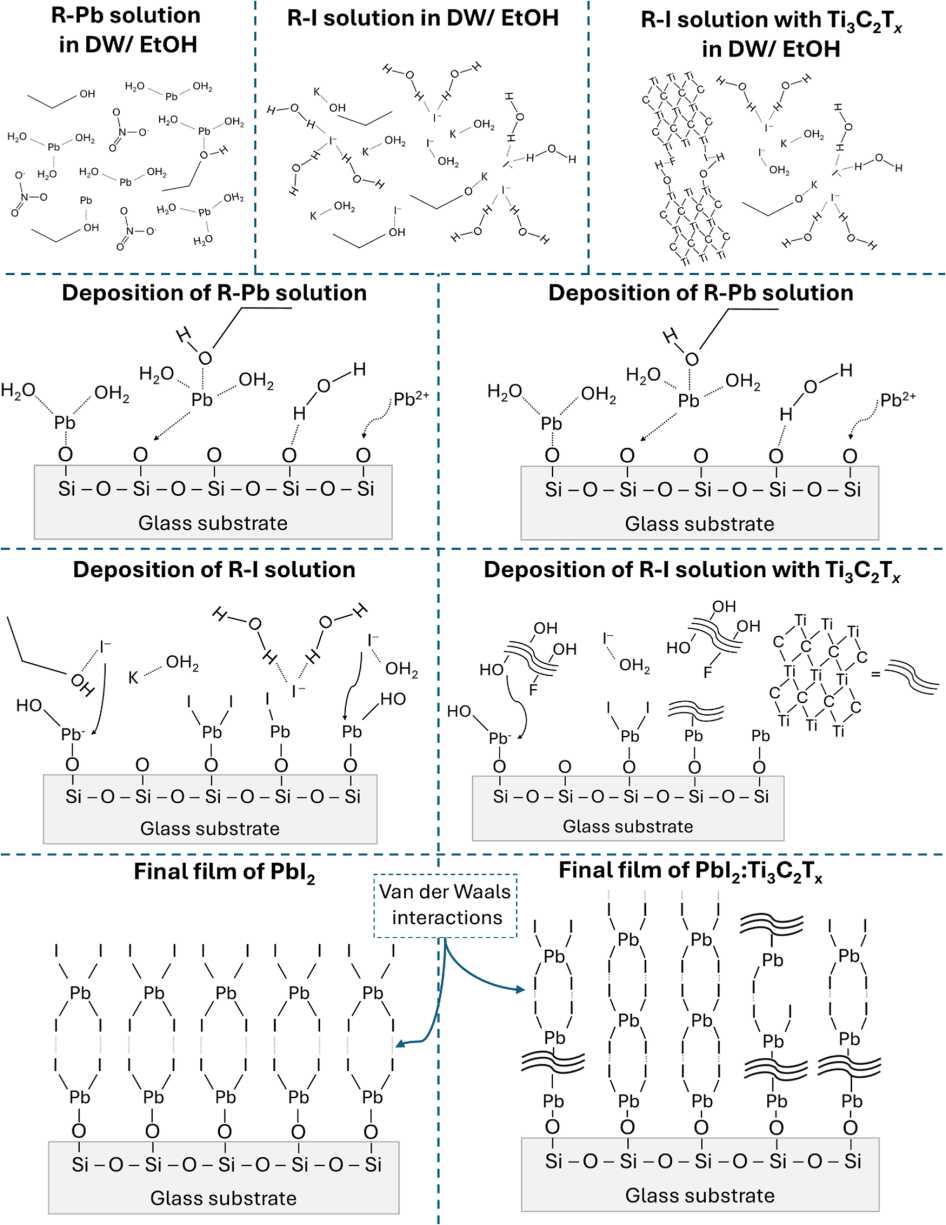
Proposed growth-mediated formation pathway of PbI_2_ and PbI_2_:Ti_3_C_2_T_*x*_ films during sequential dynamic spin-coating in a water/ethanol system. The schematic compares the pristine PbI_2_ route and the MXene-containing route. In the pristine route, the R-Pb deposition step promotes Pb-containing surface adsorption on hydroxylated glass, and the subsequent R-I deposition step promotes PbI_2_ formation. In the MXene-containing route, Ti_3_C_2_T_*x*_ introduced through the R-I solution may create MXene-derived Ti-rich contact regions that interact with Pb- and I-containing species at the growth front. These local regions modify crystallite organization, stacking-related environments, and near-surface electronic structure while preserving the 2H–PbI_2_ framework. The schematic represents an interpretation based on the correlated experimental evidence, rather than a direct atomic-scale structural model.

#### 3.7.3 Experimental Evidence Supporting the Proposed Pathway

The experimental results support the proposed pathway at several levels. First, profilometry shows that all films maintain a nearly constant average thickness across the composition series, whereas the line-scan roughness changes more clearly. This result indicates that MXene incorporation does not mainly control the total amount of deposited material, but instead modifies surface organization and local topography.

Second, SEM-EDS provides compositional support for assigning the bright clustered features to Ti-rich regions. The small-area EDS spectra collected from these bright features show markedly higher Ti contents than the corresponding large-area spectra, while Pb and I remain detectable. In several cases, the I:Pb ratios in the Ti-rich regions approach the PbI_2_-like stoichiometric value more closely than the large-area averages. These results indicate that the clustered features are not simply isolated MXene agglomerates, but MXene-derived Ti-rich regions associated with Pb–I-containing material.

Third, XRD and Raman show that the PbI_2_ framework remains structurally preserved while its local stacking-related environment changes. XRD confirms the persistence of the 2H–PbI_2_ structure across the composition series, but the (001) basal envelope evolves with MXene loading. Within the adopted phenomenological fitting model, this behavior reflects changes in the relative fitted contributions of 2H-like basal-spacing components. Raman spectroscopy supports the same interpretation because the principal PbI_2_ vibrational modes remain present, while selected modes show shifts and intensity redistribution. Together, these results indicate local stacking-related and vibrational perturbations rather than bulk lattice reconstruction.

Fourth, XPS shows that MXene incorporation modifies the local Pb–I electronic environment. The Pb 4f and I 3d core levels shift after MXene incorporation, and the Pb 4f_7/2_-I 3d_5/2_ binding-energy separation changes significantly relative to pristine PbI_2_. Because this separation would remain constant under uniform charging, its composition-dependent evolution supports a real modification of the local electrostatic and chemical environment around Pb–I bonding sites. The preservation of the dominant Pb–I chemical environment indicates that this perturbation occurs without bulk chemical transformation.

Finally, the optical and photoelectrical results remain consistent with this local perturbation model. The absorption edge remains largely preserved, indicating that MXene incorporation does not substantially reconstruct the PbI_2_ band-edge structure. However, the small near-edge shift and progressive steady-state PL quenching indicate that MXene-derived regions modify local electronic environments and promote nonradiative deactivation or interfacial carrier redistribution. The J-V/photoresponse measurements show higher absolute current density at high MXene loading, but this result is treated as a photoelectrical response rather than as evidence of a resolved transport mechanism.

#### 3.7.4 Concentration-Dependent Incorporation Regimes

The experimental trends discussed above indicate that MXene incorporation does not affect all properties in the same way across the composition series. Instead, the SEM-EDS, XRD, Raman, XPS, optical, and photoelectrical results suggest a concentration-dependent response in which local morphology, stacking-related order, electronic environment, and photoresponse evolve through distinct regimes. Therefore, the concentration-dependent behavior can be summarized as follows.

##### 3.7.4.1 Low-Loading Regime, s25 to s45

MXene incorporation primarily modifies local crystallite organization and platelet morphology. SEM shows the transition from compact granular PbI_2_ to platelet-rich surfaces, while XRD and Raman indicate that the 2H framework remains preserved with local stacking and vibrational perturbations. In this regime, MXene-derived Ti-rich regions likely act as preferential sites for Pb–I accumulation and local crystallite organization.

##### 3.7.4.2 Intermediate Perturbation Regime, Centered Near s65

The strongest stacking-related and vibrational perturbations occur in this regime. XRD shows pronounced basal-envelope modification and the lowest apparent [001] coherence length, while Raman shows mode-selective shifts consistent with local strain and stacking-related heterogeneity. SEM also shows a second morphological transition, with shorter, broader, and more locally clustered crystallite populations. This regime represents the strongest structural perturbation of PbI_2_, but it does not imply bulk phase transformation or MXene intercalation into the PbI_2_ lattice.

##### 3.7.4.3 High-Loading Regime, s100

Increasing MXene content produces more evident Ti-rich clustered regions and larger platelet aggregates. The optical and photoelectrical responses show stronger PL quenching and higher absolute current density at high loading. These effects suggest that the increased density of MXene-derived Ti-rich regions modifies interfacial electronic environments and promotes more efficient nonradiative deactivation or carrier redistribution after photoexcitation. However, because the present study does not include a dedicated transport-mechanism analysis or percolation-threshold fitting, this regime should be described as an enhanced MXene-associated photoelectrical response rather than as confirmed conductive-network formation.

These proposed regimes provide a way to connect the observed morphology, stacking-related perturbation, near-surface electronic modification, and optical/photoelectrical response. Therefore, based on the combined experimental evidence, we interpret the possible growth-mediated formation pathway of the PbI_2_-MXene films as follows:

(1) The water/ethanol sequential deposition route promotes surface-mediated PbI_2_ formation on hydroxylated glass.
(2) Ti_3_C_2_T_*x*_ introduced through the R-I solution provides MXene-derived Ti-rich contact regions with polar surface terminations.
(3) These regions may locally perturb iodide distribution and Pb–I assembly through electrostatic interactions, hydrogen bonding, and surface-termination-mediated association.
(4) MXene-derived Ti-rich regions act as preferential sites for Pb–I accumulation and local crystallite organization, leading to platelet-rich morphologies.
(5) Local interfacial coupling modifies stacking-related environments, producing basal-envelope redistribution in XRD and mode-selective Raman perturbations while preserving the 2H–PbI_2_ framework.
(6) The same local interfacial environment modifies Pb–I core-level potentials, producing statistically significant changes in the Pb 4f-I 3d binding-energy separation.
(7) At higher MXene loading, the increased density of Ti-rich domains appears to enhance PL quenching and the absolute photoelectrical response.

Thus, Ti_3_C_2_T_*x*_ modifies PbI_2_ during film formation through local interfacial organization, stacking-related perturbation, and near-surface electronic-environment modification. This model is consistent with the correlated profilometry, SEM-EDS, XRD, Raman, XPS, absorption, PL, and J-V/photoresponse results. It should be interpreted as a correlation-supported formation pathway rather than direct atomic-scale proof of the complete nucleation process. Cross-sectional TEM, high-resolution STEM-EDS, in situ growth measurements, and higher-dynamic-range synchrotron diffraction could further test the proposed nanoscale arrangement at PbI_2_/MXene contact regions. In particular, synchrotron XRD or grazing-incidence wide-angle X-ray scattering could help resolve weak nonbasal reflections and provide stronger constraints for advanced stacking-disorder models beyond the phenomenological basal-envelope analysis used here.

## 4 Conclusions

This study shows that sequential dynamic spin-coating in a water/ethanol precursor system enables in situ Ti_3_C_2_T_*x*_ incorporation into PbI_2_ films without disrupting the 2H–PbI_2_ framework. MXene incorporation does not induce a bulk phase transformation or systematic lattice expansion. Instead, it modifies the local morphology, surface organization, stacking-related environments, and near-surface Pb–I electronic environment.

The combined SEM-EDS, XRD, Raman, XPS, optical, and photoelectrical results support a proposed growth-mediated incorporation pathway in which MXene-derived Ti-rich regions associate with Pb–I-containing material and influence local crystallite organization. This interaction preserves the layered PbI_2_ structure but modifies the (001) basal-envelope line shape, selected vibrational modes, Pb 4f-I 3d binding-energy separation, PL intensity, and photoelectrical response. These correlated changes indicate that Ti_3_C_2_T_*x*_ acts as a local interfacial modifier rather than as an inert additive or a bulk lattice dopant.

Together, these results show that PbI_2_ can be treated as an engineerable precursor whose properties can be tuned through additive engineering before potential perovskite conversion, without compromising structural integrity. This perspective shifts the optimization strategy from only modifying the final halide absorber or device stack to also controlling the precursor stage itself. In practical terms, controlling the spatial distribution of MXene-derived regions may offer a route to regulate local stacking order, interfacial electrostatic environments, nonradiative deactivation pathways, and photoelectrical response while preserving the structural identity of PbI_2_. Future work should test this formation model through cross-sectional TEM, high-resolution STEM-EDS, in situ growth measurements, higher-dynamic-range synchrotron diffraction, and computational methods, while also evaluating how these precursor-stage modifications translate into perovskite conversion, film stability, and device performance.

## Supplementary Material



## Notes

Profilometry methodology; individual thickness values and propagated uncertainties; line-scan roughness analysis; representative SEM-EDS acquisition regions and spectra for large-area and small-area measurements (PDF)
