# Supplementary material for: Precursor-Stage Electronic and Stacking-Coherence Modulation of Layered PbI2 via In Situ Ti3C2T x MXene Incorporation
Source: ACS Omega. 2026 Jun 19;11(25):37528–47. doi: 10.1021/acsomega.6c02335 (PMC13325144; doi:10.1021/acsomega.6c02335)
Supplement: Supplementary file 1 [file ao6c02335_si_001.pdf]

Supporting Information

Precursor-Stage Electronic and Stacking-Coherence Modulation of Layered PbI_2 via In Situ $Ti_3C_2T_x$ MXene Incorporation

Dagoberto Cabrera-German[a,*], Luis Armando Urias-Zavala[a], Lorenzo Fuentes-Ríos[a], Guillermo Suárez-Campos[b], Jeisson Solis-Mosquera[a], Martín A. Ruiz-Molina[c], Rubén Orlando Grijalva-Saavedra[b], Manuel Quevedo-Lopez[c,a], Mérida Sotelo-Lerma[a,**]

[a] *Departamento de Investigación en Polímeros y Materiales, Universidad de Sonora, Hermosillo, Blvd Luis Encinas y Rosales s/n Sonora, 83000, México*

[b] *Departamento de Investigación en Física, Universidad de Sonora, Blvd. Luis Encinas y Rosales s/n, C.P. 83000 Hermosillo, Sonora, Mexico*

[c] *Materials Science and Engineering Department, University of Texas at Dallas, 800 West Campbell Road, Richardson, TX, 75080, United States*

*dagoberto.cabrera@unison.mx

**merida.sotelo@unison.mx

1 Film thickness and roughness determination

Film thickness was evaluated by profilometric step-height analysis. Before deposition, Kapton tape was placed at the center of the intended coating area to mask part of the glass substrate. After film deposition, the tape was removed, leaving an exposed substrate region that served as the trench reference for thickness evaluation.

For each profilometric scan, three regions were selected: the left film plateau, the trench bottom, and the right film plateau. Each region was fitted independently by linear regression. The left and right step heights were then calculated by comparing the corresponding film-plateau and trench fits at the trench edges. The uncertainty of each height was estimated from the 95% confidence bands of the fitted lines and propagated to obtain the thickness uncertainty.

Three independent samples were analyzed for each MXene loading. The reported film thickness corresponds to the average of the left and right step heights obtained from the profilometric profiles. The line-scan root-mean-square roughness, R_q, was calculated from the residuals of the left and right film-plateau regions after independently leveling each region using its fitted linear baseline.

Figure S1 shows representative profilometric traces with the selected fitting regions and their corresponding confidence bands. Table S1 summarizes the step-height and roughness values obtained for each composition.

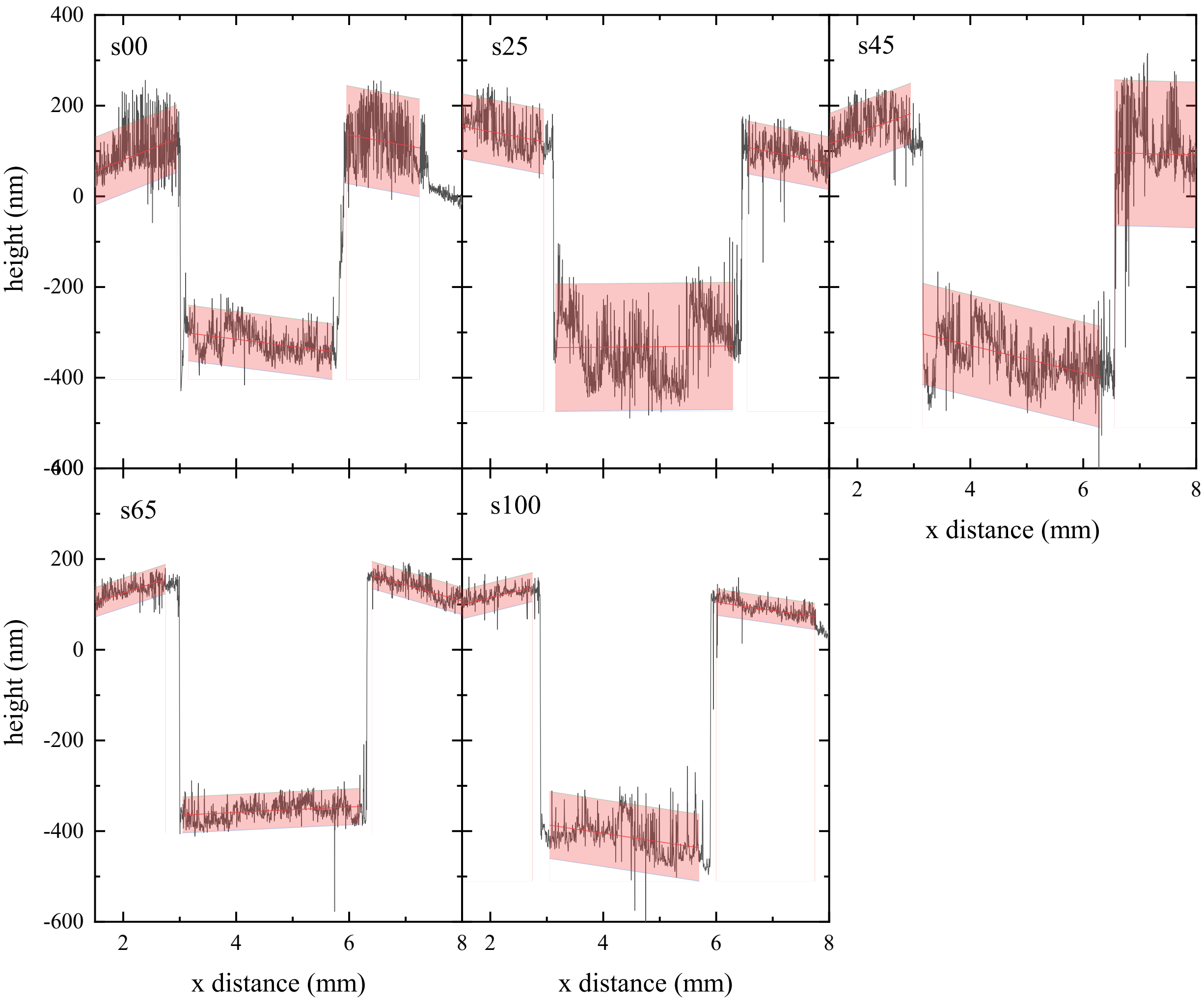

Figure S1. Representative profilometric height profiles of PbI_2 and PbI_2-MXene films showing the left film plateau, trench bottom, and right film plateau regions used for linear fitting. The trench was generated by masking the substrate with Kapton tape before deposition and removing it after film formation. Red lines indicate the fitted linear baselines, and shaded regions represent the 95% confidence bands used to estimate and propagate the uncertainty in the step-height values.

Table S1. Profilometric thickness and line-scan roughness of PbI_2-MXene films. Uncertainties were estimated from the 95% confidence bands of the linear fits and propagated accordingly.

Set	Left step-height, nm	Right step-height, nm	Mean step-height, nm	Propagated thickness uncertainty, nm	Left plateau R_q, nm	Right plateau R_q, nm	Mean line-scan R_q, nm
s00	407.9 ± 60.7	503.9 ± 66.6	455.9 ± 75.6	151.8	38.2 ± 1.5	65.7 ± 1.5	52.0 ± 8.9
s25	518.7 ± 66.5	564.3 ± 43.9	541.5 ± 56.1	129.0	39.7 ± 1.8	51.9 ± 1.4	45.1 ± 5.8
s45	468.7 ± 21.6	469.7 ± 39.7	469.2 ± 26.1	163.1	32.7 ± 1.6	44.6 ± 1.1	39.2 ± 10.1
s65	469.3 ± 57.0	496.7 ± 47.3	483.0 ± 49.0	166.4	22.9 ± 1.1	18.0 ± 1.4	20.6 ± 7.0
s100	527.3 ± 6.1	515.2 ± 35.4	521.3 ± 23.7	74.9	15.4 ± 0.8	14.5 ± 0.5	15.0 ± 2.7

2 SEM-EDS analysis of Ti-rich clustered regions

SEM-EDS analysis was performed to compare the average composition of large film regions with the local composition of bright clustered features observed in the MXene-containing samples. Large-area spectra were collected from regions of approximately 3000 μm^2, whereas small-area spectra were collected from selected bright clustered features with areas ranging from approximately 2 to 120 μm^2, depending on the feature size.

The semi-quantitative analysis was restricted to Pb, I, and Ti signals and renormalized to these elements. Other detected elements were excluded from this comparison because they may include contributions from the glass substrate, adventitious surface species, or instrumental/background signals. This normalization allows direct comparison of the PbI_2- and MXene-relevant elemental contributions in large-area and localized regions.

The small-area spectra collected from bright clustered features show markedly higher Ti fractions than the corresponding large-area spectra, confirming that these features correspond to Ti-rich regions associated with MXene-derived domains. Pb and I signals remain detectable in these Ti-rich regions, indicating that the clustered features are not isolated MXene agglomerates but are associated with Pb-I-containing material.

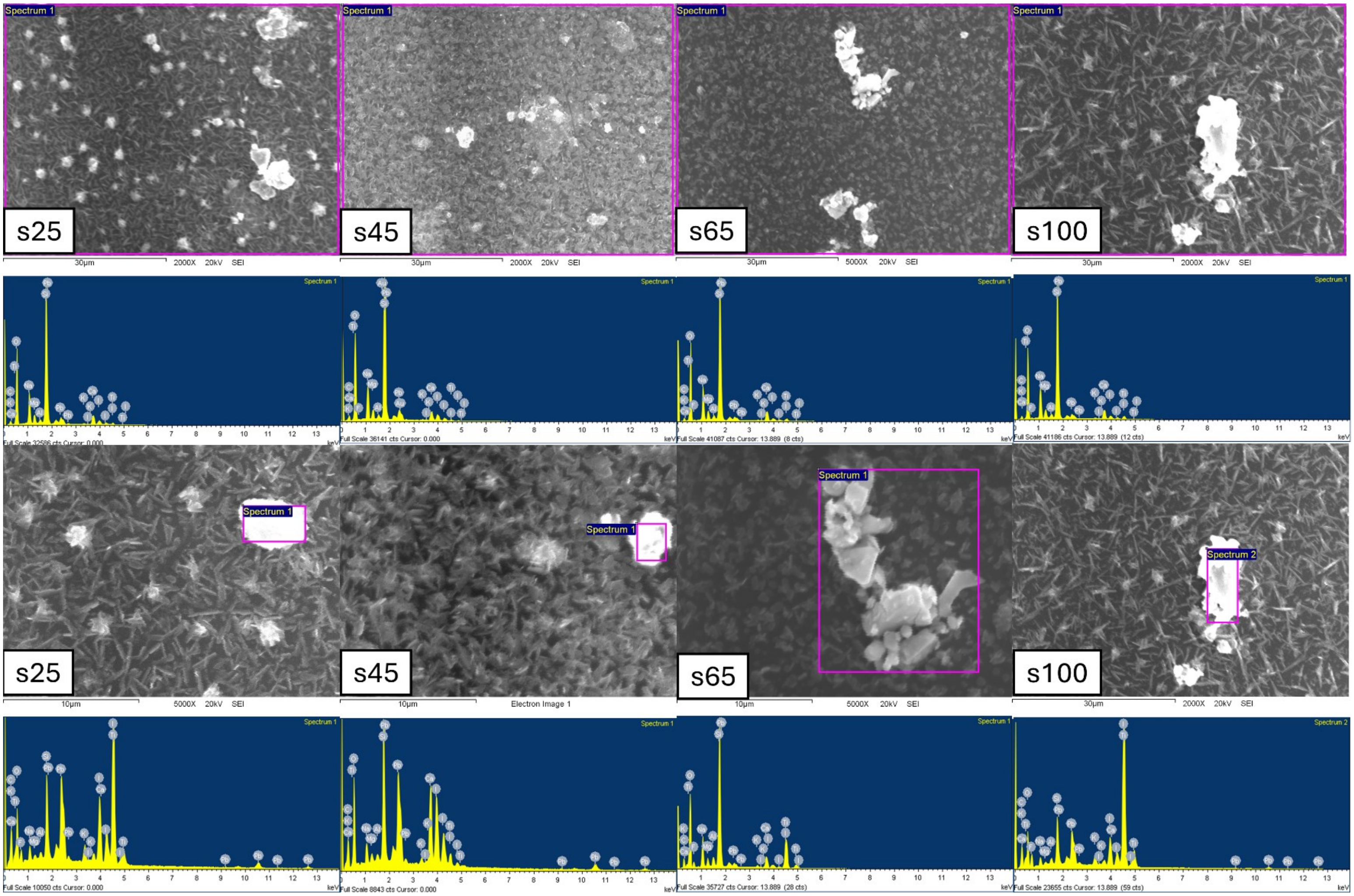

Figure S2. Representative SEM micrographs of PbI2-MXene films showing the regions used for SEM-EDS analysis. Large-area spectra were collected over approximately 3000 μm^2 to estimate the average composition of the inspected film region. Small-area spectra were collected from bright clustered features with areas ranging from approximately 2 to 120 μm^2. The corresponding EDS spectra confirm enrichment of Ti in the bright clustered regions.
